# Supplementary material for: PNPLA3 I148M variant links to adverse metabolic traits in MASLD during fasting and feeding
Source: JHEP Rep. 2025 May 10;7(8):101450. doi: 10.1016/j.jhepr.2025.101450 (PMC12269597; doi:10.1016/j.jhepr.2025.101450)
Supplement: Multimedia component 1 [file mmc1.pdf]

# PNPLA3 I148M variant links to adverse metabolic traits in MASLD during fasting and feeding

Lina Jegodzinski, Lorena Rudolph, Darko Castven, Friedhelm Sayk, Ashok Kumar Rout, Bandik Föh, Laura Hölzen, Svenja Meyhöfer, Andrea Schenk, Susanne N. Weber, Monika Rau, Sebastian M. Meyhöfer, Jörn M. Schattenberg, Marcin Krawczyk, Andreas Geier, Alvaro Mallagaray, Ulrich L. Günther, Jens U. Marquardt

Table of contents

Supplementary methods……………………………………………………………………2

Fig. S1……………………………………………………………………………………5

Fig. S2……………………………………………………………………………………7

Fig. S3……………………………………………………………………………………8

Fig. S4……………………………………………………………………………………10

Fig. S5……………………………………………………………………………………11

Fig. S6……………………………………………………………………………………12

Table S1…………………………………………………………………………………..14

Table S2…………………………………………………………………………………...15

Supplementary references……………………………………………………………16

## Supplementary methods

### NMR-Proteometabolomics

*Sample preparation*

Samples were prepared following a previously described standard operating procedure (SOP)[1]. Briefly, frozen serum aliquots were thawed at room temperature for a maximum of 30 minutes. Serum and NMR buffer (75 mM sodium phosphate buffer pH 7.40, 20% deuterium oxide, 4.8 mM 3-trimethyl-silyl-[2,2,3,3-$^{2}H_4$]propionic acid or TMSP-d4 and 0.04% $NaN_3$) were 1:1 homogenized, and 600 µL of the well-mixed sample were transferred to a 5 mm NMR tube. The tubes were cooled at 279 K in an automated SampleJet™ until measurement. Spectra were acquired within a maximum of 24 h from sample preparation.

*NMR measurements*

All NMR experiments were performed on a Bruker Avance III HD 600 MHz NMR spectrometer equipped with a TXI room temperature probe and a Bruker SampleJet™ automatic sample exchanger with sample storage set at 6 °C. All experiments were acquired at 310 K. Temperature precision, quantification accuracy, water suppression performance and gradient profiles were tested and calibrated when required on a daily basis following Bruker´s commercial SOPs. All NMR experiments were acquired in full automation. In all the cases the carrier was placed at the center of the water signal and the receiving gain was set to 90.5. **$^{1}$H-NOESY** experiments were acquired using the standard pulse sequence *noesygppr1d* from Bruker library with 32 scans (4m 3s), 96k points, 30 ppm spectral width and 4 s inter-scan delay. **$^{1}$H-CPMG** experiments were acquired using the standard pulse sequence *cpmgpr1d* from Bruker library with 32 scans

(4m 18s), 96k points, 30 ppm spectral width, 4 s inter-scan relaxation delay, 0.3 ms echo time for a total of 78 ms $T_2$ relaxation time.

For the processing of these two experiments, FIDs were zero filled to 128k points and multiplied by an exponential decay window function to obtain a line broadening of 0.3 Hz. After Fourier transform, spectra were calibrated to the TMSP-d4 signal at 0 ppm and zero order phase corrected. Bruker Quantification in Plasma/Serum (B.I.Quant-PS 2.0.0) and Bruker IVDr Lipoprotein Subclass Analysis (B.I.-LISA) were used to automatically quantify 39 metabolites (+2 technical additives) and 112 lipoprotein parameters, including very-low-density lipoprotein (VLDL), intermediate-density lipoprotein (IDL), low-density lipoprotein (LDL), and high-density lipoprotein (HDL). In addition, several subfractions of triglycerides (TG), cholesterol (CH), free cholesterol (FC), phospholipids (PL), and apolipoproteins (Apo) were calculated. The LDL was categorized into six subclasses based on specific density ranges: LDL-1 (1.019–1.031), LDL-2 (1.031–1.034), LDL-3 (1.034–1.037), LDL-4 (1.037–1.040), LDL-5 (1.040–1.044), and LDL-6 (1.044–1.063).

NMR glycoslation profiles from circulating acute-phase proteins were derived from **selTOCSY** and **JEDI-PGPE** experiments[2,3]. **SelTOCSY** spectra were acquired using the standard pulse sequence *selmlgp.2* from Bruker library with 32k points and 30 ppm spectral width, 0.3 ms inter-scan relaxation delay and 50 ms TOCSY mixing time. Selective irradiation was achieved using a Gaussian-shaped Gaus1.1000 pulse. Frequency for selective pulse, pulse length and number of scans were as follows: When centered at 3.7 ppm (selTOCSY-3.7), the pulse had a length of 8 ms and 600 scans (13m 8s) were acquired. When centered at 4.3 ppm (selTOCSY-4.3), the pulse had a length of 9 ms and 312 scans (6m 52s) were acquired. When centered at 4.85 ppm (selTOCSY-4.85), the pulse length was set to 25 ms and 600 scans (13m 18s) were acquired. FIDs

were zero-filled to 128k points and multiplied by an exponential decay window function to obtain a line broadening of 0.3 Hz. **JEDI-PGPE** experiments were acquired using the standard pulse sequence *zgpezf.ivdr* from Bruker library with 32 scans (2m 19s), 96k points, 30 ppm spectral width, 1 s inter-scan relaxation delay, the gradient shaped pulses were set to 2.5 ms and 80% gradient strength (from a maximum nominal gradient strength of 48.15 G/cm), the total $T_2$ relaxation time was 110 ms and the Eddy current time delay was set to 5 ms. In both cases spectra were Fourier transformed, calibrated, zero-order phase corrected, baseline corrected, and lineshape fitted to quantify the phosphatidylcholine concentration from the SPC signal (extracted from PGPE-JEDI experiment) and from SPE-1 and SPE-2 signals (extracted from selTOCSY-3.7 and selTOCSY-4.3, respectively), and to obtain glycosylation profiles[4].

Additionally, **Fig. S6** shows examples of lineshape fitting of representative NMR spectra.

## Supplementary figures

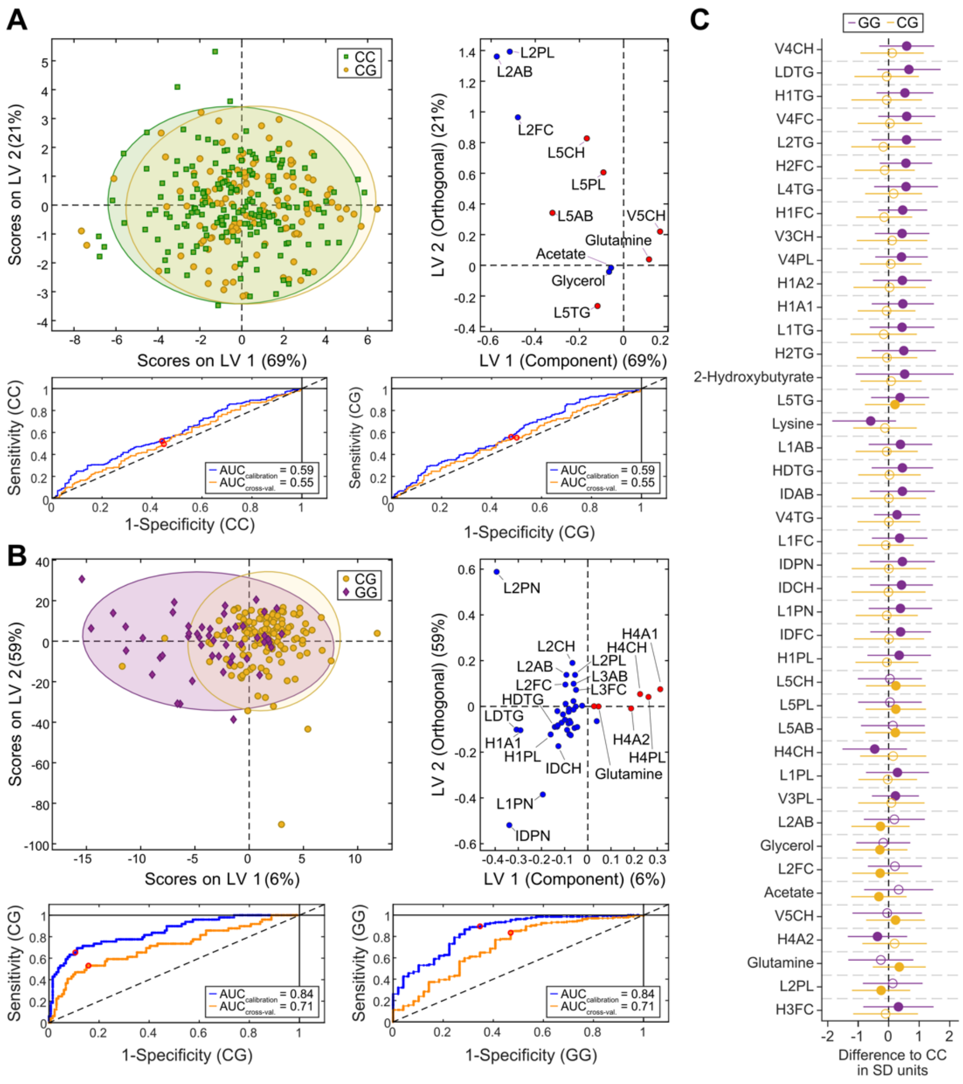


**Fig. S1: NMR metabolite and lipoprotein analysis based on *PNPLA3* genotype.** (A) Distribution of *PNPLA3* CG *vs.* CC carriers and (B) *PNPLA3* CG *vs.* GG carriers based on the O-PLS-DA of metabolites and lipoproteins of parameters with significant differences between the groups (*q*-value <0.05), loading plot of significant parameters (red and blue indicate increase and decrease in CG or GG carriers, respectively) and corresponding ROC curves**.** (C) Forest plot showing significant changes in metabolites

and lipoproteins of either CG or GG carriers, both with CC carriers as reference (middle line shows reference mean, while circles on horizontal axes show changes scaled by standard deviations (SD)). Statistically significant differences are indicated by filled circles (determined using a false discovery rate (FDR) of 5%).

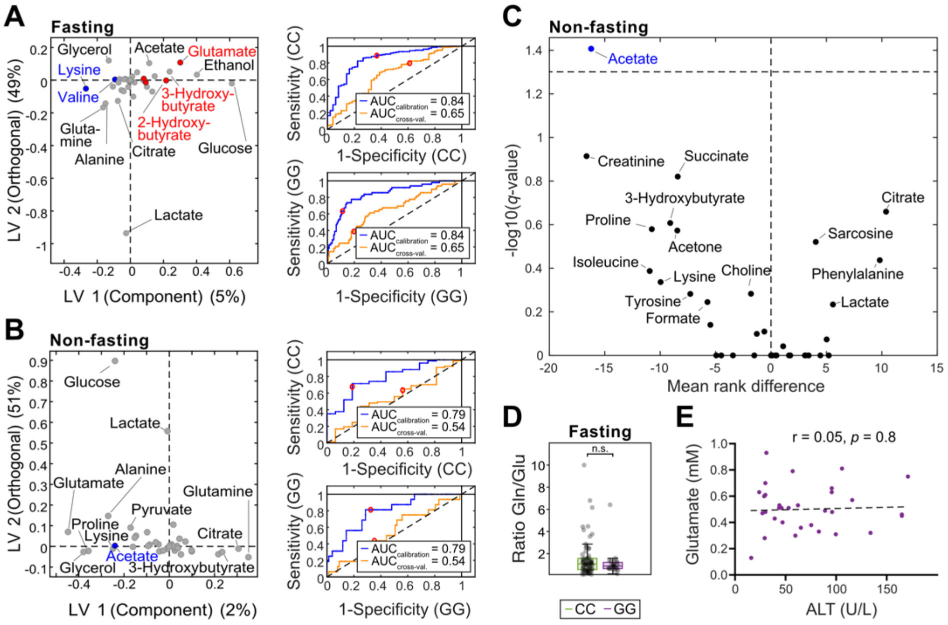


**Fig. S2: Fasting and non-fasting metabolite differences between *PNPLA3* GG and CC genotypes.** (A) Loading plots and corresponding ROC curves of O-PLS-DAs of fasting and (B) non-fasting metabolites between *PNPLA3* GG and CC carriers. (C) Vulcano plot illustrating non-fasting metabolites of *PNPLA3* GG carriers compared to CC carriers. (D) Box plot of fasting glutamine (Gln)-to-glutamate (Glu) ratio between groups (bars represent SD and bold lines within box plots represent medians; Mann-Whitney U test; false discovery rate (FDR): 5%). (E) Correlation between glutamate and ALT levels in fasting PNPLA3 GG carriers (two-tailed Pearson correlation test).

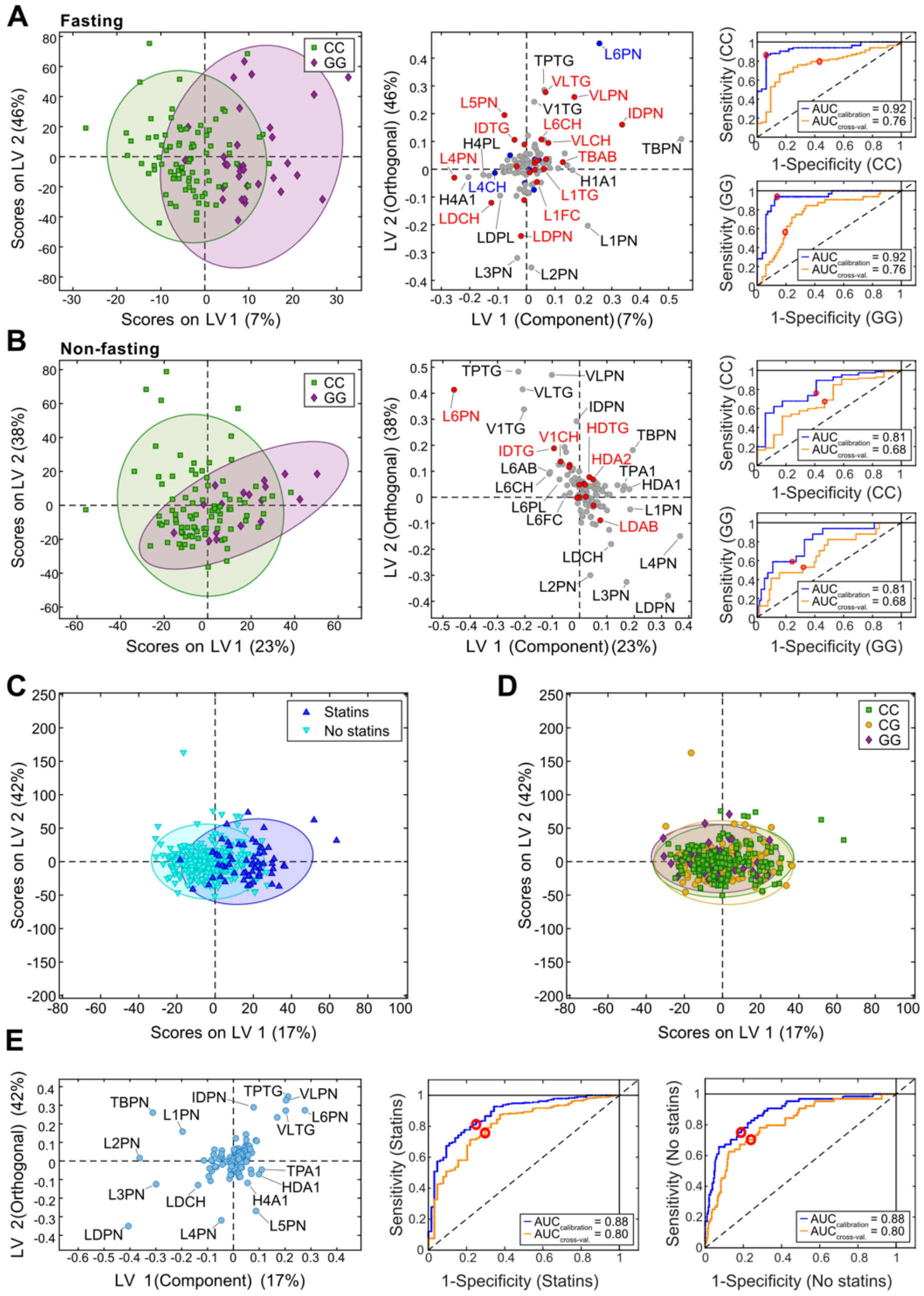


**Fig. S3: Remarkable variation in fasting and non-fasting lipoproteins in homozygous *PNPLA3* GG carriers.** (A) Separation of *PNPLA3* GG *vs.* CC carriers using O-PLS-DA of fasting and (B) non-fasting lipoproteins, loading plots and corresponding ROC curves**.** (C) Separation of patients with and without statin use based on O-PLS-DA

of lipoproteins and (D) distribution of genotypes (CC, CG and GG) within this separation.

(E) Loading plot and corresponding ROC curves of O-PLS-DA.

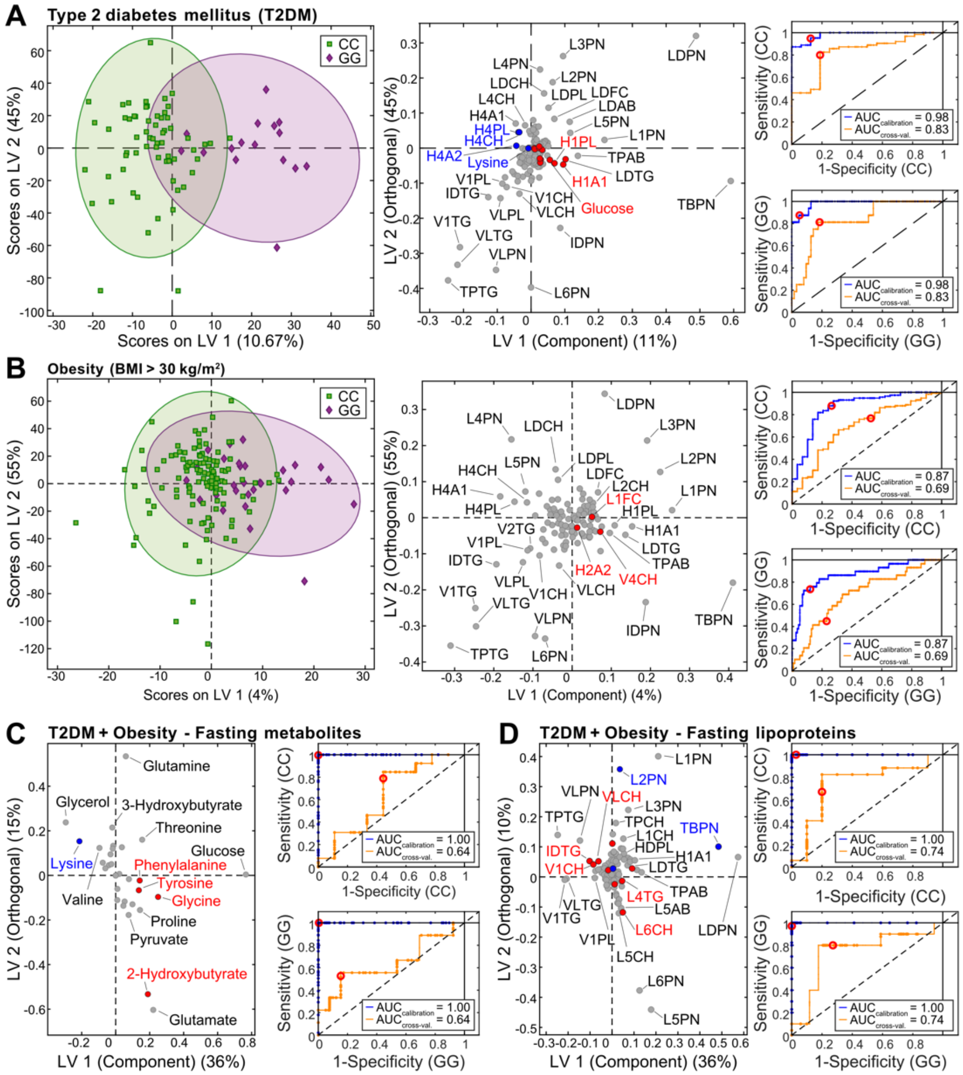


**Fig. S4: Type 2 diabetes mellitus (T2DM) and obesity amplify *PNPLA3* GG and CC differentiation.** (A) Distribution of *PNPLA3* GG *vs.* CC carriers with T2DM and in (B) of *PNPLA3* GG *vs.* CC carriers with obesity using O-PLS-DA of metabolites and lipoproteins, loading plots highlighting significant parameters ($q$-value <0.05) and corresponding ROC curves. (C) Loading plots and corresponding ROC curves of fasting metabolites and (D) fasting lipoproteins between *PNPLA3* GG and CC carriers with T2DM and obesity.

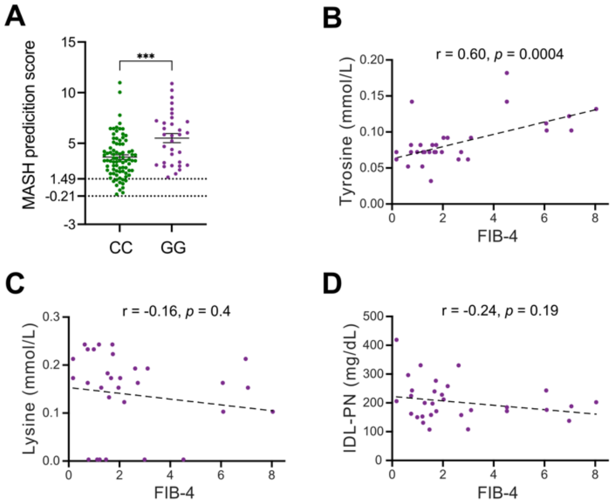


**Fig. S5: Correlation between individual parameters and FIB-4.** (A) Differences in MASH prediction score between fasting *PNPLA3* GG and CC carriers (mean ± standard error of the mean (SEM)). (B) Correlations between tyrosine, (C) lysine and (D) Intermediate-density lipoprotein particle number (IDL-PN) and FIB-4 values in fasting *PNPLA3* GG carriers (two-tailed Pearson correlation test). ***p <0.001 (Mann-Whitney U test).

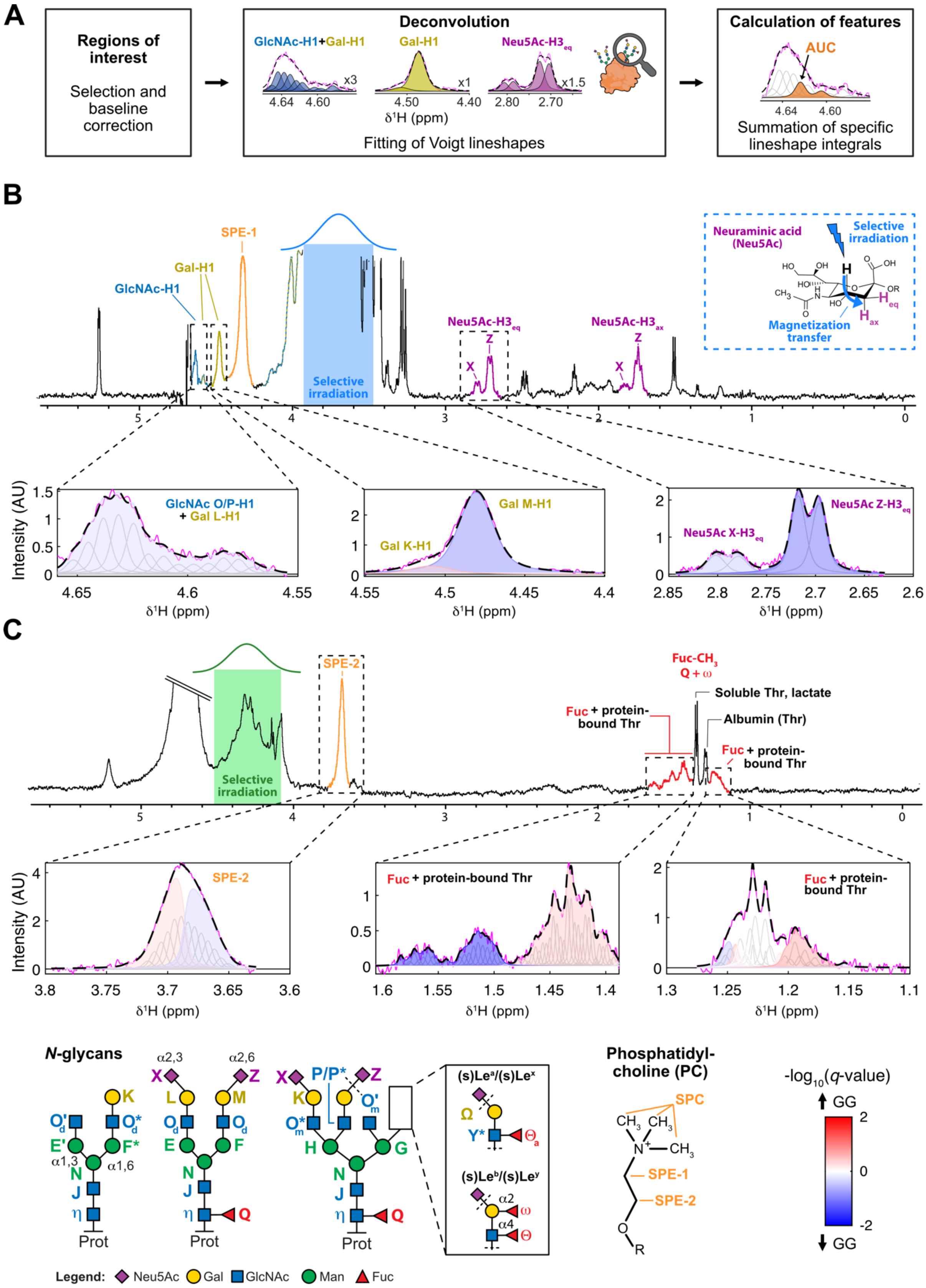
A
Regions of interest
Selection and baseline correction
Deconvolution
GlcNAc-H1+Gal-H1
Gal-H1
Neu5Ac-H3eq
Fitting of Voigt lineshapes
Calculation of features
AUC
Summation of specific lineshape integrals
B
SPE-1
Gal-H1
GlcNAc-H1
Selective irradiation
Neu5Ac-H3eq
Neu5Ac-H3ax
Neuraminic acid (Neu5Ac)
Selective irradiation
Magnetization transfer
Intensity (AU)
GlcNAc O/P-H1 + Gal L-H1
Gal M-H1
Gal K-H1
Neu5Ac Z-H3eq
Neu5Ac X-H3eq
δ1H (ppm)
C
SPE-2
Fuc-CH3
Q + ω
Soluble Thr, lactate
Albumin (Thr)
Fuc + protein-bound Thr
Selective irradiation
N-glycans
Prot
(s)Lea/(s)Lex
(s)Leb/(s)Ley
Phosphatidyl-choline (PC)
SPC
SPE-1
SPE-2
-log10(q-value)
↑ GG
↓ GG
Legend: Neu5Ac Gal GlcNAc Man Fuc

**Fig. S6:** Examples of lineshape fitting of representative NMR spectra. (A) Workflow for serum glycoprotein profiling using lineshape fitting. (B) Lineshape fitting of selTOCSY-3.7 experiment and (C) selTOCSY-4.3 experiment (cite our Anal. Chem. here). Differences in the area under the fitted curves (AUCs) between *PNPLA3* CC and GG carriers are shown as $-\log_{10}(q\text{-values})$ in red and blue, whereby red indicates an increase in GG and blue indicates a decrease. The inlet in B demonstrates the principle of selTOCSY, where selected glycan and phosphatidylcholine protons are irradiated to transfer magnetization over three bonds to the target protons, exemplified by selective irradiation of Neu5Ac-H4 and transfer to Neu5Ac-H3.

## Supplementary Table

**Table S1. Post-hoc analysis of significant patient characteristics based on *PNPLA3* genotype.**

| | ***PNPLA3* rs738409** | | |
|---|---|---|---|
| ***p* value[1]** | **CC *vs.* CG** | **CC *vs.* GG** | **CG *vs.* GG** |
| AST, U/L | 0.137 | **0.002** | 0.142 |
| ALT, U/L | **0.002** | **0.001** | 0.790 |
| Platelet count, x10^9/L | 0.762 | **0.001** | **0.020** |
| FIB-4 | 1.000 | **0.001** | **<0.001** |

ALT, alanine aminotransferase; AST, aspartate aminotransferase.

[1]ANOVA with Bonferroni's adjustment for pairwise comparisons was used. Bold *p* values denote statistical significance at the p <0.05 level.

**Table S2. Comparison of Lysine and IDL-particle number (PN) concentrations between fasted *PNPLA3* GG and CC carriers.**

| Variable | Source | df | Mean square | *F* value | *p* value[1] | partial η² | *r* value |
|---|---|---|---|---|---|---|---|
| **Lysine** (mmol/L) | AST | 1 | 0.006 | 0.775 | 0.380 | 0.007 | 0.08 |
| | ALT | 1 | 0.009 | 1.217 | 0.272 | 0.011 | 0.11 |
| | Platelet count | 1 | 0.014 | 1.919 | 0.169 | 0.017 | 0.13 |
| | **PNPLA3 genotype** | 1 | 0.023 | 3.187 | 0.077 | 0.028 | 0.17 |
| **IDL-PN** (mg/dL) | AST | 1 | 1393 | 0.323 | 0.571 | 0.003 | 0.05 |
| | **ALT** | 1 | 17722 | 4.112 | **0.045** | **0.036** | **0.19** |
| | Platelet count | 1 | 1117 | 0.259 | 0.612 | 0.002 | 0.05 |
| | **PNPLA3 genotype** | 1 | 24962 | 5.792 | **0.018** | **0.050** | **0.22** |

IDL, intermediate-density lipoprotein; ALT, alanine aminotransferase; AST, aspartate aminotransferase; df, degrees of freedom.

[1]ANCOVA (adjusted for ALT, AST, platelet count and *PNPLA3* genotype (GG *vs.* CC)) was used. Bold *p* values denote statistical significance at the p <0.05 level.

## Supplementary references


1. **Schmelter, F, Föh, B**, Mallagaray, A, *et al.* Metabolic and Lipidomic Markers Differentiate COVID-19 From Non-Hospitalized and Other Intensive Care Patients. *Frontiers in Molecular Biosciences* **8,** (2021).

2. Lodge, S, Nitschke, P, Kimhofer, T, *et al.* Diffusion and Relaxation Edited Proton NMR Spectroscopy of Plasma Reveals a High-Fidelity Supramolecular Biomarker Signature of SARS-CoV-2 Infection. *Anal. Chem.* **93,** 3976–3986 (2021).

3. Rudolph, L, Krellmann, R, Castven, D, *et al.* Fast NMR-Based Assessment of Cancer-Associated Protein Glycosylations from Serum Samples. *Anal. Chem.* accepted (2025).

4. Mallagaray, A, Rudolph, L, Lindloge, M, *et al.* Towards a Precise NMR Quantification of Acute Phase Inflammation Proteins from Human Serum. *Angewandte Chemie International Edition* **62,** e202306154 (2023).
