# Supplementary material for: PNPLA3 I148M variant links to adverse metabolic traits in MASLD during fasting and feeding
Source: JHEP Rep. 2025 May 10;7(8):101450. doi: 10.1016/j.jhepr.2025.101450 (PMC12269597; doi:10.1016/j.jhepr.2025.101450)
Supplement: Multimedia component 2 [file mmc2.docx]

**JHEP Reports**

**CTAT methods**

Tables for a “Complete, Transparent, Accurate and Timely account” (CTAT) are now mandatory for all revised submissions. The aim is to enhance the reproducibility of methods.

- Only include the parts relevant to your study
- Refer to the CTAT in the main text as ‘Supplementary CTAT Table’
- Do not add subheadings
- Add as many rows as needed to include all information
- Only include one item per row

**If the CTAT form is not relevant to your study, please outline the reasons why:**

|  |
| --- |

- 1. **Antibodies**

| **Name** | **Citation** | **Supplier** | **Cat no.** | **Clone no.** |
| --- | --- | --- | --- | --- |
|  |  |  |  |  |

- 1. **Cell lines**

| **Name** | **Citation** | **Supplier** | **Cat no.** | **Passage no.** | **Authentication test method** |
| --- | --- | --- | --- | --- | --- |
|  |  |  |  |  |  |

- 1. **Organisms**

| **Name** | **Citation** | **Supplier** | **Strain** | **Sex** | **Age** | **Overall n number** |
| --- | --- | --- | --- | --- | --- | --- |
|  |  |  |  |  |  |  |

- 1. **Sequence based reagents**

| **Name** | **Sequence** | **Supplier** |
| --- | --- | --- |
|  |  |  |

- 1. **Biological samples**

| **Description** | **Source** | **Identifier** |
| --- | --- | --- |
| EDTA blood samples | Human participants | N/A |
| Serum blood samples | Human participants | N/A |

- 1. **Deposited data**

| **Name of repository** | **Identifier** | **Link** |
| --- | --- | --- |
|  |  |  |

- 1. **Software**

| **Software name** | **Manufacturer** | **Version** |
| --- | --- | --- |
| SPSS Statistics | IBM, Armonk, NY | 29.0.2.0 (20) for Mac OS X |
| GraphPad Prism | GraphPad Software, La Jolla, CA | 10.3.1 (464) for Mac OS X |
| Matlab | MathWorks, Natick, MA | 2022a |
| PLS_Toolbox software package | Eigenvector Research, Inc., Manson, WA, USA | 9.1 |

- 1. **Other (*e.g*. drugs, proteins, vectors etc.)**

| DNeasy Blood and Tissue Kit | Qiagen |  |
| --- | --- | --- |
| TaqMan assay | Thermo Fisher | C_7241_10 |

- 1. **Please provide the details of the corresponding methods author for the manuscript:**

| Prof. Dr. med. Jens U. Marquardt (jens.marquardt@uksh.de)  Prof. Dr. rer. nat. Ulrich L. Günther (ulrich.guenther@uni-luebeck.de) |
| --- |

**2.0 Please confirm for randomised controlled trials all versions of the clinical protocol are included in the submission. These will be published online as supplementary information.**

|  |
| --- |
