# Supplementary material for: PNPLA3 I148M variant links to adverse metabolic traits in MASLD during fasting and feeding
Source: JHEP Rep. 2025 May 10;7(8):101450. doi: 10.1016/j.jhepr.2025.101450 (PMC12269597; doi:10.1016/j.jhepr.2025.101450)
Supplement: Multimedia component 4 [file mmc4.pdf]

PNPLA3 I148M variant links to adverse metabolic traits in MASLD during fasting and feeding☆

Lina Jegodzinski[1], Lorena Rudolph[2,3], Darko Castven[1], Friedhelm Sayk[1], Ashok Kumar Rout[2], Bandik Föh[1], Laura Hölzen[1], Svenja Meyhöfer[1,4], Andrea Schenk[5], Susanne N. Weber[6], Monika Rau[7], Sebastian M. Meyhöfer[1,8], Jörn M. Schattenberg[6], Marcin Krawczyk[9], Andreas Geier[7], Alvaro Mallagaray[2], Ulrich L. Günther[2,*,†], Jens U. Marquardt[1,*,†]

JHEP Reports **2025**. vol. 7 | 1–12

Check for updates

Background & Aims: The *PNPLA3* rs738409 polymorphism is the most abundant genetic risk factor associated with progression of metabolic dysfunction-associated steatotic liver disease (MASLD) to steatohepatitis (MASH) and fibrosis. Although fasting and feeding affect *PNPLA3* expression, molecular insights into the pathophysiological influence remain scarce.

Methods: We analyzed 353 serum samples of patients with MASLD from two German tertiary centers using nuclear magnetic resonance (NMR)-proteometabolomics. Patients were stratified by *PNPLA3* rs738409 C>G genotype: 'CC', 'CG', and 'GG'. Metabolites, lipoproteins, and glycoproteins were assessed based on fasting status.

Results: *PNPLA3* GG displayed a distinct metabolic profile, with notable alterations between fasting and non-fasting states. During the latter, GG carriers showed lower levels of VLDL-1, reflecting impaired triglyceride (TG) efflux from hepatocytes. Following an overnight fast, GG carriers exhibited higher tricarboxylic acid cycle metabolites and ketone bodies, overall indicating increased β-oxidation likely attributed to lower *PNPLA3* expression, facilitating unrestricted adipose triglyceride lipase activity and consecutive increased hepatic TG secretion. In addition, the ketogenic amino acid lysine, critical for mitochondrial carnitine transport, was significantly reduced (GG 0.14 ± 0.09 mM *vs.* CC 0.18 ± 0.08 mM, $q = 0.015$). Consistently, TGs were enriched in LDL and HDL particles, and an increased number of intermediate-density lipoproteins emerged as a distinct marker in fasted GG carriers (GG 202.9 ± 68.2 mg/dl *vs.* CC 160.8 ± 65.6 mg/dl, $q = 0.007$). These metabolic changes were enhanced in patients with type 2 diabetes mellitus and/or obesity.

Conclusions: Our findings suggest a dichotomous pattern of increased hepatic lipid storage during feeding and excessive lipid oxidation during fasting, which exceeds metabolic capacity, inducing cellular toxicity in *PNPLA3* GG carriers. This interplay fuels a detrimental fasting/non-fasting cycle, thus pointing to the need for preventive strategies.

© 2025 The Authors. Published by Elsevier B.V. on behalf of European Association for the Study of the Liver (EASL). This is an open access article under the CC BY license (http://creativecommons.org/licenses/by/4.0/).

Introduction

Metabolic dysfunction-associated steatotic liver disease (MASLD) affects approximately 30% of the global population, with obesity and type 2 diabetes mellitus (T2DM) as major risk factors.[1,2] The current concept considers a stepwise disease progression from simple steatosis to steatohepatitis (MASH), fibrosis, and cirrhosis, which can ultimately result in hepatocellular carcinoma development.[3] MASLD also increases the risk of cardiovascular disease and other chronic diseases, as well as cancers.[4] The course of the disease is influenced by a complex interplay of genetics, epigenetics, lifestyle factors, body composition, and the gut microbiome.[5]

The most common genetic risk factor associated with MASLD/MASH is the rs738409 (c.444C>G) polymorphism in the patatin-like phospholipase domain-containing 3 (*PNPLA3)* gene, present in 30–50% of individuals. This variant results in an isoleucine-to-methionine substitution at position 148 of the protein (p.I148M).[6] PNPLA3 is a membrane-bound protein located on hepatic lipid droplets, where it hydrolyzes triglycerides (TGs) in hepatocytes and retinol esters in stellate cells.[7,8] The PNPLA3 148M variant accelerates MASLD progression and increases liver-related mortality, among others.[9] Specifically, the 148M variant binds to the lipase co-activator ABHD5 (CGI-58) and sequesters it away from adipose triglyceride lipase (ATGL), preventing its activation and leading to reduced lipid remodeling in hepatocytes.[10,11] In addition, the PNPLA3 148M protein effectively escapes ubiquitination, resulting in its accumulation on lipid droplets, whereby it fuels a vicious cycle.[12] Although the mechanisms that affect hepatic fibrogenesis are not fully understood, it may increase oxidative

☆ Given their role as Associate Editor, Jörn M. Schattenberg had no involvement in the peer review of this article and had no access to information regarding its peer review. Full responsibility for the editorial process for this article was delegated to the Associate Editor Maja Thiele and Co-Editor Jacob George.

* Corresponding authors. Addresses: Department of Medicine I, University Hospital Schleswig-Holstein, Ratzeburger Allee 160, 23538 Lübeck, Germany (J.U. Marquardt); Institute of Chemistry and Metabolomics, University of Lübeck, Ratzeburger Allee 160, 23562 Lübeck, Germany (U.L. Günther).
E-mail addresses: jens.marquardt@uksh.de (J.U. Marquardt), ulrich.guenther@uni-luebeck.de (U.L. Günther).
† These are joint last authors.

https://doi.org/10.1016/j.jhepr.2025.101450

and endoplasmic reticulum stress through enhanced lipotoxicity.[13] In addition, PNPLA3 I148M carriers were shown to have increased β-oxidation and an elevated hepatic mitochondrial redox state during fasting, likely attributed to unrestricted ATGL activity.[14] Thus, it seems plausible that differences in fasting status of patients may affect the impact of *PNPLA3* genotypes.

To gain further insight into the metabolic changes associated with the PNPLA3 I148M variant in MASLD, we performed multicenter NMR-based proteometabolomic analysis in 353 patients with MASLD stratified by *PNPLA3* status. Our data revealed distinct alterations in homozygous *PNPLA3* GG carriers, particularly pronounced in patients following overnight fasting. Specifically, GG carriers displayed elevated fasting ketone bodies and tricarboxylic acid (TCA) cycle metabolites, as well as reduced lysine levels, which is crucial for mitochondrial carnitine transport. Consistently, we observed a higher TG load in LDL and HDL subtypes and identified a significantly increased number of intermediate-density lipoprotein (IDL) particles in fasted *PNPLA3* GG carriers. In contrast, GG carriers tended to have reduced VLDL-1 in the non-fasted state, reflecting impaired hepatic lipid secretion.

Our study demonstrates that the *PNPLA3* I148M variant disrupts metabolic perturbations and confirms the critical impact of fasting and feeding cycles on *PNPLA3* expression,[15] emphasizing its functional and pathogenic role during MASLD/MASH progression. These findings highlight the importance of preventive interventions to counteract this maladaptive metabolic interplay and provide new avenues for biomarker identification.

Materials and methods

Patient cohort

Between August 2022 and August 2023, a total of 353 patients with MASLD were included from two tertiary centers: the Department of Medicine II at the University Hospital Würzburg, Bayern, and the Department of Medicine I at the University Hospital Schleswig-Holstein, Lübeck (Table 1). Hepatic steatosis was identified by imaging or biopsy. All participants met the Delphi consensus criteria for the identification of MASLD, fulfilling at least one metabolic criterion.[16] Patients with self-reported daily alcohol consumption exceeding 20 g for women and 30 g for men, or with other known liver diseases, were excluded.[17] Serum and EDTA blood samples were collected for NMR-proteometabolomics and genotyping, and clinical and demographic parameters were recorded during routine examinations. Patients were randomly seen either after an overnight fast or under non-fasting conditions. Notably, the proportion of patients enrolled after overnight fasting was 48.8% for *PNPLA3* CC (n = 83), 48.5% for CG (n = 65), and 65.3% for GG carriers (n = 32). Importantly, patients receiving

Table 1. Patient characteristics based on *PNPLA3* genotype.

	PNPLA3 rs738409			
	**CC (n = 170)**	**CG (n = 134)**	**GG (n = 49)**	***p* value***
Age (years)	53 ± 12	50 ± 15	51 ± 14	0.139
Sex, female/male (%)	54/46	46/54	53/47	0.310
BMI (kg/m^2)	36.4 ± 9.1	35.5 ± 8.9	33.2 ± 7.1	0.069
Presence of obesity (%)	68.2	66.4	59.2	0.498
Presence of diabetes (%)	37.6	28.6	32.7	0.249
Presence of hypertension (%)	65.7	65.4	51.0	0.146
Presence of dyslipidemia (%)	47.1	52.2	36.7	0.174
Medication				
Metformin (%)	30.3	24.2	18.8	0.217
Insulin (%)	12.1	14.4	8.5	0.566
SGLT-2 inhibitors (%)	10.6	8.2	6.1	0.573
GLP-1 RA (%)	14.1	15.7	8.2	0.426
Statin use (%)	20.0	18.0	14.9	0.716
Laboratory				
HbA1c (%)	6.1 ± 1.0	6.0 ± 0.9	6.0 ± 1.5	0.872
Total cholesterol (mg/dl)	193.3 ± 48.9	193.5 ± 44.1	194.5 ± 54.5	0.991
LDL-C (mg/dl)	116.5 ± 42.9	120.6 ± 37.5	114.7 ± 49.0	0.625
HDL-C (mg/dl)	49.2 ± 16.2	46.5 ± 11.0	51.8 ± 16.0	0.077
Triglyceride (mg/dl)	190.5 ± 118.7	186.4 ± 103.4	161.2 ± 96.1	0.324
Creatinine (µmol/L)	79.6 ± 17.7	80.4 ± 16.8	79.6 ± 22.1	0.960
AST (U/L)	**35.1** ± **17.3**	**40.0** ± **24.7**	**47.0** ± **21.1**	**0.002**
ALT (U/L)	**46.1** ± **26.2**	**61.2** ± **49.1**	**68.7** ± **43.1**	**< 0.001**
Platelet count (× 10^9/L)	**251** ± **69**	**242** ± **61**	**211** ± **74**	**0.002**
FIB-4	**1.3** ± **1.2**	**1.2** ± **0.9**	**2.1** ± **1.9**	**< 0.001**
FibroScan, CAP (dB/m)	330 ± 61	335 ± 48	327 ± 40	0.567
FibroScan, median (kPa)	8.3 ± 5.6	9.1 ± 5.4	9.6 ± 6.0	0.344
Patients with liver biopsy, n (%)	45 (26.5)	37 (27.6)	12 (24.5)	
NAS	3.4 ± 1.8	3.8 ± 1.8	3.5 ± 2.0	0.515
Steatosis grade	1.5 ± 0.7	1.6 ± 0.8	1.6 ± 0.7	0.915
Ballooning grade	1.0 ± 0.8	1.2 ± 0.8	0.9 ± 0.7	0.417
Inflammation grade	0.8 ± 0.7	1.1 ± 0.7	1.1 ± 0.9	0.335
Fibrosis stage	1.0 ± 1.1	1.2 ± 1.2	1.6 ± 1.1	0.313

Data are presented as mean ± SD or %. *The Chi-square test was used for categorical variables, and ANOVA (with Bonferroni's adjustment for pairwise comparisons) was used for continuous variables. Bold *p* values denote statistical significance at the *p* <0.05 level. ALT, alanine aminotransferase; AST, aspartate aminotransferase; CAP, controlled attenuation parameter; GLP-1RA, glucagon-like peptide-1 receptor agonist; NAS, NAFLD activity score; SGLT-2, sodium-glucose cotransporter-2.

sodium-glucose cotransporter-2 (SGLT-2) inhibitors, which are known to exacerbate ketogenesis,[18] were excluded from further analysis of fasting metabolites.

All participants provided written informed consent after being fully informed about the study. The protocol was approved by the ethics committees in Würzburg (AZ 96/12 and 188/17) and Lübeck (AZ 22-180) and was conducted in accordance with the Declaration of Helsinki.

Genotyping

Genotyping of the *PNPLA3* variant rs738409 (p.I148M) was conducted centrally at the genetic laboratory (GastroLabor) of the Department of Medicine II, Saarland University Medical Center, Homburg, Germany. DNA was extracted from peripheral blood mononuclear cells using the DNeasy Blood and Tissue Kit (Qiagen (Hilden, Germany)). DNA concentrations were quantified using a NanoDrop spectrophotometer. The variant was genotyped using TaqMan assay (ID: C_7241_10, Thermo Fisher (Waltham, MA, USA)).[19] Based on their *PNPLA3* rs738409 C>G genotype, patients were classified into three subgroups: *PNPLA3* ‘CC’, ‘CG’, or ‘GG’.

NMR-proteometabolomics

Samples were prepared following a previously described standard operating procedure (SOP).[20] NMR spectra were acquired within a maximum of 24 h after sample preparation. All NMR experiments were performed on a Bruker Avance III HD 600 MHz NMR spectrometer equipped with a TXI room temperature probe and a Bruker SampleJet™ automatic sample exchanger with sample storage set at 6 °C. All experiments were conducted at 310 K. Temperature precision, quantification accuracy, water suppression performance, and gradient profiles were tested and calibrated daily following Bruker’s SOPs. All NMR experiments were conducted in full automation.

Bruker Quantification in Plasma/Serum (B.I.Quant-PS 2.0.0) and Bruker IVDr Lipoprotein Subclass Analysis (B.I.-LISA, Bruker Corporation (Billerica, MA, USA)) were used to automatically quantify 39 metabolites (+2 technical additives) and 112 lipoprotein parameters, including VLDL, IDL, LDL, and HDL. In addition, several subfractions of TGs, cholesterol (CH), free cholesterol (FC), phospholipids (PL), and apolipoproteins (Apo) were calculated. The LDL was categorized into six subclasses based on specific density ranges: LDL-1 (1.019–1.031), LDL-2 (1.031–1.034), LDL-3 (1.034–1.037), LDL-4 (1.037–1.040), LDL-5 (1.040–1.044), and LDL-6 (1.044–1.063). Details on recent strategies for the quantification of lipoprotein parameters from NMR spectra can be found in the bibliography.[21,22]

NMR glycosylation profiles from circulating acute-phase proteins were derived from the selTOCSY and JEDI-PGPE experiments.[23,24] In both cases, spectra were Fourier-transformed, calibrated, zero-order phase-corrected, baseline-corrected, and line shape-fitted to quantify phosphatidylcholine concentration from the supramolecular phosphatidylcholine composite signal (SPC; extracted from the JEDI-PGPE experiment) and to obtain glycosylation profiles, as reported elsewhere.[25] Further methodological details, including examples on line shape fitting of representative NMR spectra, are described in the Supplementary Materials and methods.

Statistical analysis

Statistical analyses of clinical data were performed using SPSS Statistics Version 29.0.2.0 (20) for Mac OS X (IBM, Armonk, NY, USA) and GraphPad Prism 10.3.1 (464) for Mac OS X (GraphPad Software, La Jolla, CA, USA). Clinical variables with more than 15% missing values were excluded, unless otherwise indicated. Data are presented as either mean ± SD or percentage (%). Comparisons between the three groups were made using ANOVA (with Bonferroni’s adjustment for pairwise comparisons) and the Chi-square test.

Statistics of serum concentrations of metabolites and lipoprotein parameters were conducted using in-house scripts in Matlab 2022a (MathWorks, Natick, MA, USA). For the analysis of glycosylation patterns and phosphatidylcholine concentration (SPC signal), statistically significant differences between groups were determined from areas under the curves corresponding to SPC and acute-phase glycoprotein signals. All line shape integrals of the same spectral feature with a q value (corrected p value) <0.01 were summed up and used as statistically significant features. The Mann–Whitney U and Kruskall–Wallis tests with Dunn’s multiple comparisons were used to compare two and three groups, respectively. The false discovery rate method of Benjamini, Krieger, and Yekutieli was applied to correct for multiple testing (Q = 0.05).

For supervised orthogonal partial least squares discriminant analysis (O-PLS-DA), the data were preprocessed with orthogonal signal correction, Pareto scaling, and mean centering. Venetian blinds were used as a cross-validation method with 10 data splits, one sample per blind, and 10% left-out data. O-PLS-DA was performed in the PLS_Toolbox software package, version 9.1 (Eigenvector Research, Inc., Manson, WA, USA) running in Matlab 2022a.

Two-tailed Pearson correlations were calculated to assess the correlation between clinical variables and metabolomics parameters. Analysis of covariance (ANCOVA) was used to compare lysine and IDL concentrations between fasted *PNPLA3* GG and CC carriers, adjusting for appropriate covariates such as alanine aminotransferase (ALT), aspartate aminotransferase (AST), platelet count, and *PNPLA3* genotype (CC *vs*. GG). A two-tailed p value <0.05 was considered statistically significant for all analyses.

Results

Patient characteristics

A total of 353 patients with MASLD were included in this observational study (Fig. 1A). Frequencies of the *PNPLA3* rs738409 genotypes were comparable to the prevalence reported in the literature.[26] In detail, 48% of patients were homozygous for the CC genotype, 38% were heterozygous (CG) and 14% were homozygous carriers of the GG variant (Fig. 1B). Minor deviation from Hardy–Weinberg equilibrium was observed, showing a slight predominance of GG over CG carriers, which may be attributed to the specialized nature of these institutions. Baseline clinical characteristics stratified by *PNPLA3* genotype are summarized in Table 1.

Patients of all *PNPLA3* genotypes exhibited comparable age, sex distribution, and diabetes prevalence. Although

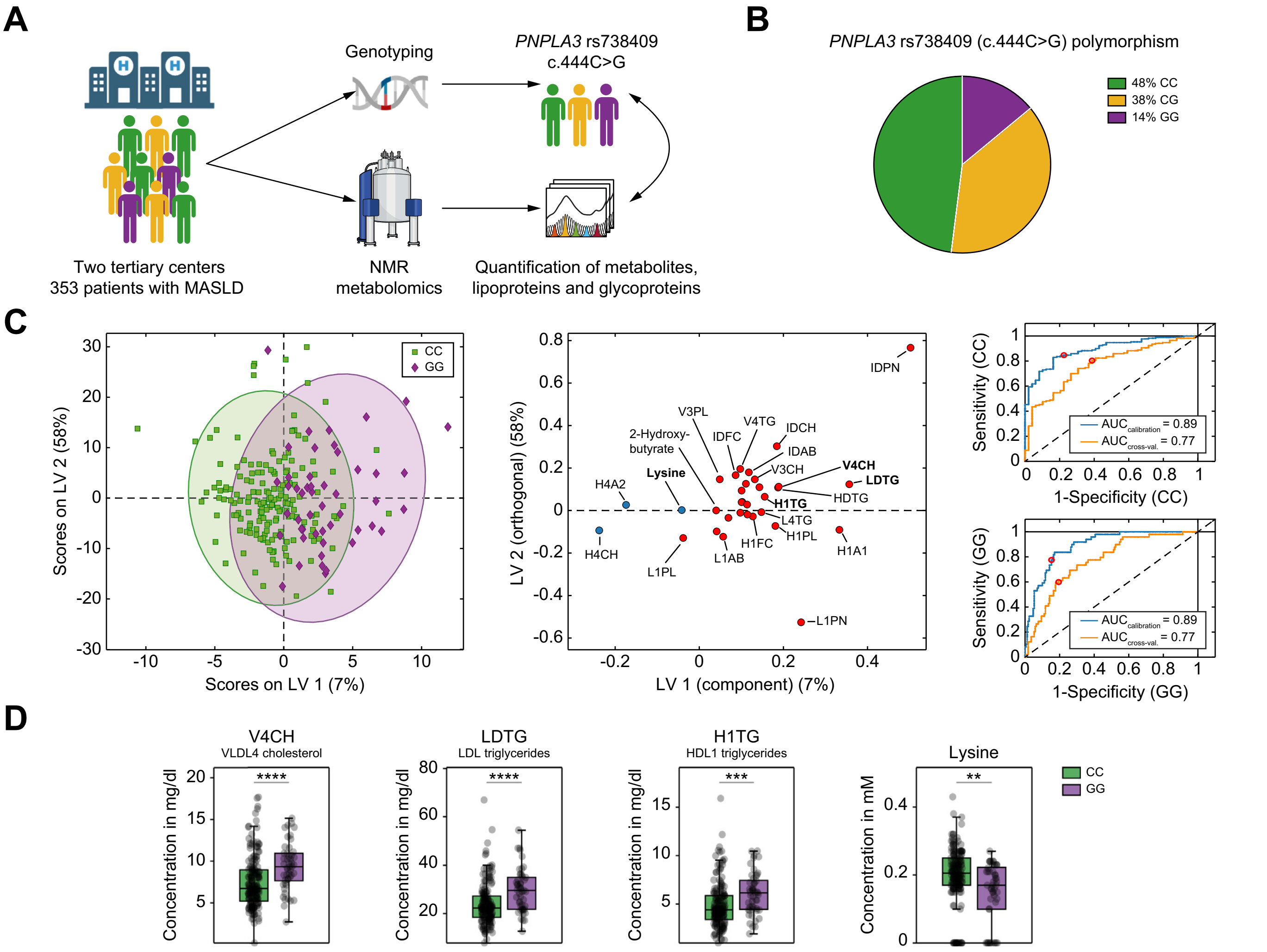

Fig. 1. Distinct NMR metabolite and lipoprotein profiles in *PNPLA3* GG carriers. (A) Schematic overview of the study design. (B) Frequencies of *PNPLA3* rs738409 C>G genotypes. (C) Distribution of *PNPLA3* GG *vs*. CC carriers based on the O-PLS-DA of metabolites and lipoproteins using parameters with significant differences between the groups (*q* value <0.05), loading plot of significant parameters (red and blue indicate increase and decrease in GG carriers, respectively), and corresponding ROC curves. (D) Box plots of the three most significant lipoproteins and the most significant metabolite between groups (bars represent SD, and bold lines within the box plots represent medians). Levels of significance: $^{**}q$ <0.01; $^{***}q$ <0.001; $^{****}q$ <0.0001 (Mann–Whitney *U* test; FDR 5%). FDR, false discovery rate; NMR, nuclear magnetic resonance; O-PLS-DA, orthogonal partial least squares discriminant analysis; PNPLA3, patatin-like phospholipase domain-containing protein; ROC, receiver operating characteristic.

PNPLA3 GG carriers showed a trend toward lower BMI and less frequent obesity compared with non-carriers, these differences remained without statistical significance. Similarly, the prevalence of hypertension and dyslipidemia did not differ between genotypes. Importantly, intake of antidiabetic medications and statins was consistent across all groups.

Patients with CG and GG genotypes showed significantly higher plasma levels of ALT. In addition, homozygous GG carriers presented with elevated AST, lower platelet counts, and higher FIB-4 scores within the range of intermediate fibrosis risk (mean ± SD, 2.05 ± 1.92 in GG, 1.21 ± 0.89 in CG, and 1.32 ± 1.24 in CC). However, although vibration-controlled transient elastography (VCTE) measurements showed a trend toward greater liver stiffness in GG (9.57 ± 5.9 4 kPa) compared with CG (9.07 ± 5.42 kPa) and CC carriers (8.34 ± 5.62 kPa), these differences were not statistically significant. Similarly, no differences were observed in controlled attenuation parameter (CAP) values. Among the patients who underwent liver biopsy (approximately 25%), no significant histological changes were detected. Overall, these results confirm an increased susceptibility of *PNPLA3* CG and GG carriers to develop more advanced liver disease.

Differences in NMR metabolite and lipoprotein profiles stratified by *PNPLA3* genotype

Analysis of NMR metabolite and lipoprotein profiles in patients with MASLD revealed profound differences according to the different *PNPLA3* genotypes. These differences were most noticeable between homozygous *PNPLA3* GG carriers and non-carriers (CC) ($AUC_{cross\text{-}val.}$ = 0.77), with two metabolites and 30 lipoproteins showing significant differences (Fig. 1C and Fig. S1C). Among the key findings, GG carriers exhibited higher levels of small CH-rich VLDL-4 (V4CH), TG-rich LDL (LDTG),

and HDL-1 (H1TG). In addition to changes in lipoprotein subtypes, the metabolic analysis indicated a significant reduction in the amino acid lysine (Fig. 1D).

Interestingly, heterozygous carriers of the variant (CG) did not show substantial differences in metabolite or lipoprotein profiles compared with *PNPLA3* CC carriers ($AUC_{cross\text{-}val.}$ = 0.55). However, a clear separation was observed between CG and homozygous GG carriers ($AUC_{cross\text{-}val.}$ = 0.71) (Fig. S1A–C), revealing a unique signature of homozygous carriers of the *PNPLA3* rs738409 (p.I148M) variant.

Overnight fasting reveals metabolite differences

For a detailed characterization of metabolic alterations in relation to the fasting state, we next focused on metabolite differences between homozygous *PNPLA3* GG and CC carriers during fasting and non-fasting states. Interestingly, we identified several metabolites that displayed significant differences after an overnight fast (Fig. 2A), which were not observed in patients in the non-fasting state (Fig. 2B). Compared with the metabolic analyses obtained in the total cohort, the overall number of significantly different metabolites was higher in *PNPLA3* GG carriers than in CC carriers in fasted (n = 6) *vs.* non-fasted (n = 1) states (Fig. 2C and Fig. S2C).

Notably, in the fasting state, *PNPLA3* GG carriers exhibited significantly higher levels of succinate, a central intermediate, and glutamate, a product of the TCA cycle, compared with *PNPLA3* CC carriers. They also showed a tendency toward increased ketone bodies, including 3-hydroxybutyrate and acetone, along with higher concentrations of 2-hydroxybutyrate, known to be released as a by-product of glutathione synthesis. The ketogenic amino acid lysine, which is also involved in the mitochondrial carnitine shuttle, was significantly lower in *PNPLA3* GG carriers. In contrast, the amino acid tyrosine,

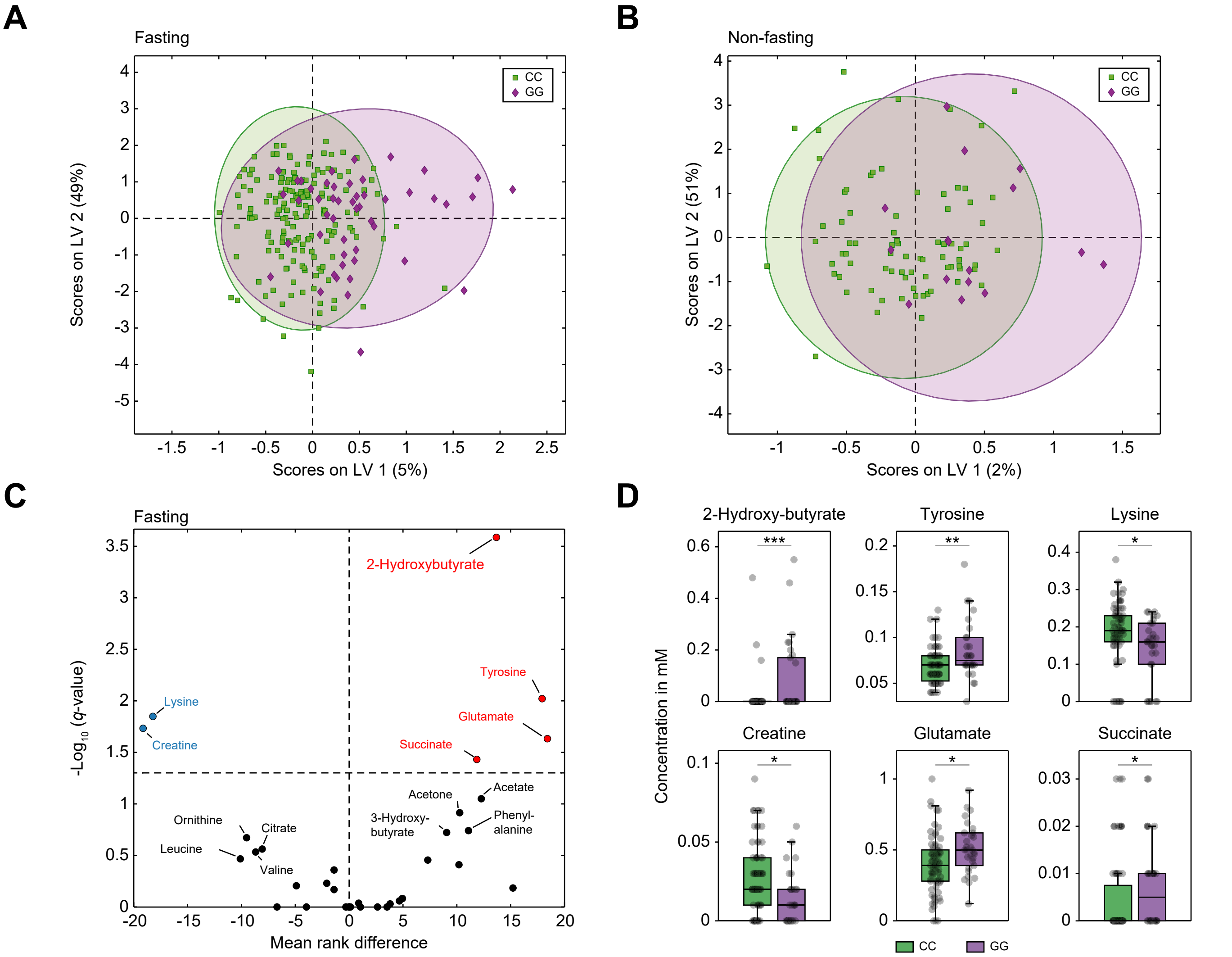

Fig. 2. Metabolite differences in the fasting and non-fasting states in *PNPLA3* GG compared with CC carriers. Distribution of *PNPLA3* GG *vs.* CC carriers based on the O-PLS-DA of metabolites in patients (A) after an overnight fast (GG n = 32/CC n = 83) and (B) in a non-fasting condition (GG n = 17/CC n = 87). (C) Volcano plot illustrating fasting metabolites of *PNPLA3* GG carriers compared with CC carriers. (D) Significant fasting metabolites between groups (bars represent SD, and bold lines within the box plots represent medians). Levels of significance: **q* <0.05; ***q* <0.01; ****q* <0.001 (Mann–Whitney *U* test; FDR = 5%). FDR, false discovery rate; O-PLS-DA, orthogonal partial least squares discriminant analysis; PNPLA3, patatin-like phospholipase domain-containing protein.

precursor of catecholamines and thyroxine, was elevated in these patients. Another notable aspect was the reduced levels of creatine in *PNPLA3* GG carriers (Fig. 2C and D).

In contrast, many of the previously elevated metabolites, including succinate and 3-hydroxybutyrate, were lower in non-fasted *PNPLA3* GG carriers (Fig. 2B and Fig. S2C). Taken together, these results suggest that the distinct metabolic signature in *PNPLA3* GG carriers is primarily evident after an overnight fast.

Lipoprotein signatures differentiate *PNPLA3* GG carriers by higher fasting IDL particle number

To assess whether lipoproteins show the same pattern as metabolites in relation to fasting and non-fasting states, we analyzed the distribution of lipoprotein subtypes between *PNPLA3* GG and CC carriers under the different conditions. Here, lipoprotein profiles revealed a distinct separation between homozygous *PNPLA3* GG carriers and non-carriers (CC) in both fasted (Fig. 3A and Fig. S3A) and non-fasted states (Fig. 3B and Fig. S3B).

In the fasting state, *PNPLA3* GG carriers showed a high TG load across multiple LDL subtypes, with additional elevations in TG within large HDL particles (HDL-1 and HDL-2). Smaller HDL particles (HDL-3/HDL-4), in contrast, exhibited significantly reduced PL and CH levels. GG carriers also had higher levels of VLDL-3 and VLDL-4. Importantly, focusing on the total particle number, the number of IDL particles, as a transient degradation product of VLDL and an intermediate stage to LDL particles, showed a significantly higher concentration in *PNPLA3* GG compared with CC carriers (Fig. 3C and D).

In the non-fasting state, significant alterations in lipoprotein subfractions were observed. Specifically, TG-rich LDL-4 and PL- and CH-rich VLDL-5 were significantly elevated in homozygous *PNPLA3* GG carriers (Fig. 3C and E). Furthermore, there was a noticeable trend toward a reduction in VLDL-1 particles, including VLDL-TG (V1TG), PL (V1PL), and CH (V1CH) (Fig. 3E), indicating a low rate of conversion and export of TGs from hepatocytes.

Interestingly, the presence of the GG variant has also been associated with diminished protective effects of statins against MASH in clinical trials.[26] However, our analysis found no influence of statin use on the distribution of lipoproteins across *PNPLA3* genotypes (Fig. S3C–E).

These results demonstrate that despite the absence of significant differences in clinical lipoprotein parameters, we identified remarkable changes in lipoprotein subgroups in both fasting and non-fasting states, with a significantly higher fasting IDL particle number in patients who were homozygous for the *PNPLA3* rs738409 (p.I148M) variant.

T2DM and obesity amplify *PNPLA3*-related metabolic changes

Several lines of evidence suggest that in patients with either T2DM or obesity (BMI >30 kg/m^2), *PNPLA3* is significantly upregulated by insulin resistance. Therefore, changes in the metabolome between homozygous *PNPLA3* GG carriers and non-carriers (CC) might be more pronounced. Our results are concordant with these observations and revealed profound differences between these two groups in patients with either T2DM or obesity, regardless of fasting state (Fig. S4A and B). Consistently, patients with concomitant T2DM and obesity showed the strongest separation between genotypes, irrespective of fasting state ($AUC_{cross\text{-}val.}$ = 0.90) (Fig. 4A).

Further analysis of the fasting state revealed significant differences between GG and CC genotypes in metabolite (Fig. 4B) and lipoprotein (Fig. 4C) profiles in the combined comorbidities as well as in the single groups (Fig. 4D). In *PNPLA3* GG carriers with T2DM, levels of phenylalanine, a precursor of tyrosine, and glycine were higher. In patients with obesity carrying the GG genotype, glutamate levels were markedly elevated, showing some slight differences between subgroups. Importantly, although 2-hydroxybutyrate and tyrosine were significantly higher, lysine levels were consistently reduced in all metabolic risk groups in *PNPLA3* GG compared with CC carriers (Fig. 4D).

Similarly, differences in fasting lipoproteins between genotypes were detected in both the subgroups with T2DM and obesity. However, changes in HDL subfractions were more pronounced in *PNPLA3* GG patients with obesity than in those with T2DM. In contrast, differences in LDL subfractions were predominantly observed in GG carriers with concomitant T2DM and obesity, with elevated levels of L4TG and L5TG also found primarily in the GG subgroup with T2DM, each compared with CC carriers (Fig. 4D).

Notably, *PNPLA3* GG carriers with diabetes and obesity had significantly lower VLDL-1 lipids even in the fasted state compared with CC carriers (Fig. 4D), highlighting the impact of the *PNPLA3* rs738409 (p.I148M) variant in these patients at high metabolic risk.

PNPLA3-driven glycoprotein differences are not linked to acute-phase inflammatory markers

Analysis of combined acute-phase inflammation glycoprotein and phosphatidylcholine profiles (SPC) revealed several differences between *PNPLA3* GG and CC carriers (Fig. 5A and C). Specifically, GG carriers exhibited a considerably lower GlycA to GlycB ratio (Fig. 5D). GlycA and GlycB are composite biomarkers that reflect both the protein levels and glycosylation states of acute-phase proteins. Specifically, GlycA originates from N-acetyl methyl groups of neuraminic acid (also known as sialic acid), and GlycB arises from N-acetyl methyl groups from N-acetylglucosamine (GlcNAc) moieties decorating acute-phase inflammation proteins.[27,28] Therefore, the lower GlycA to GlycB ratios in GG carriers suggest alterations in the glycosylation patterns or relative abundances of the selected acute-phase proteins.

In contrast, the SPC signal envelope, which is mainly associated with PL in LDL and HDL particles,[29] and the GlycAB to SPC ratio showed no differences between genotypes (Fig. 5D), indicating that acute-phase inflammation and lipid-associated inflammatory markers were not different between CC and GG carriers, suggesting that the differences obtained may be independent of the inflammatory state of the patients.

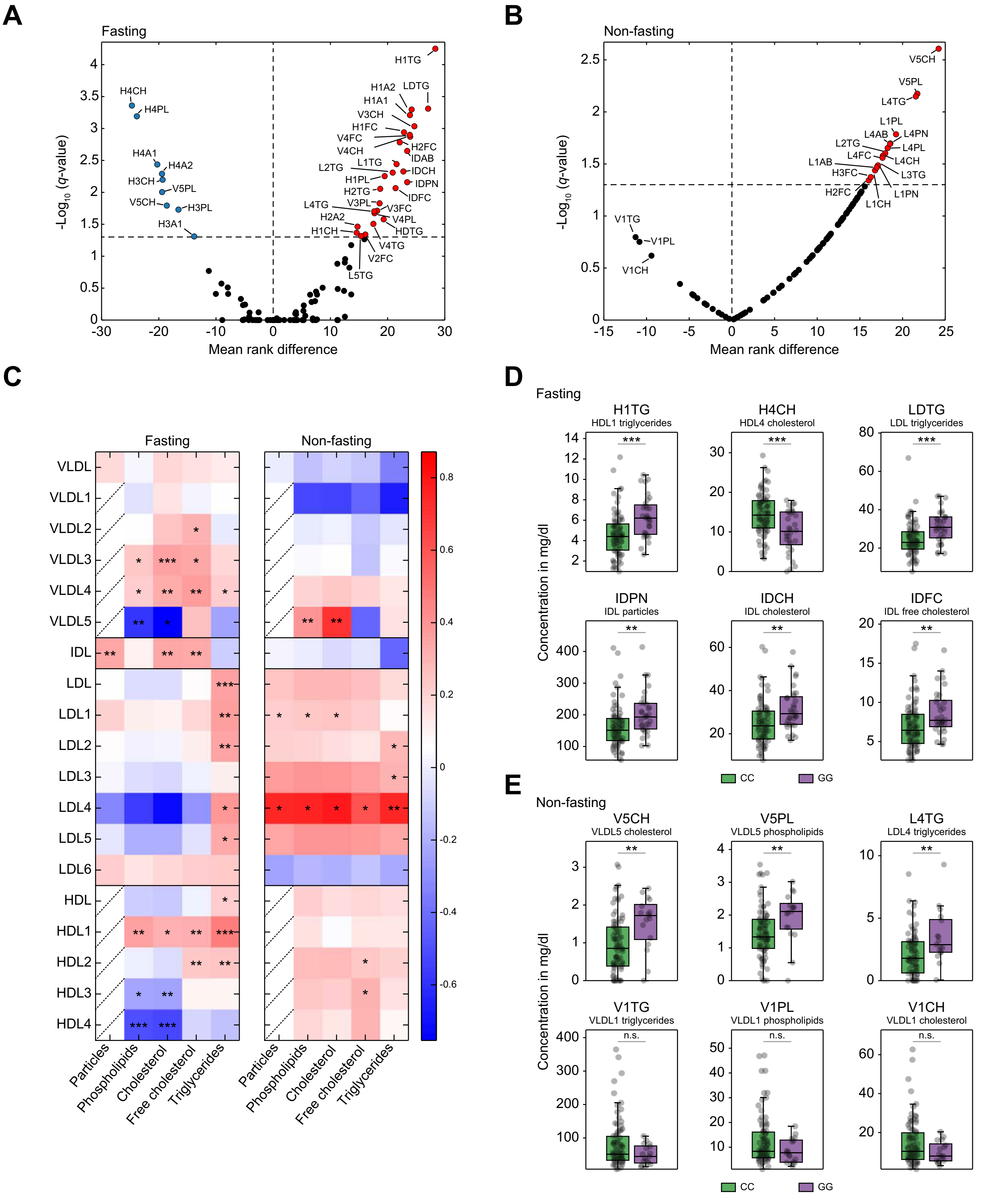

Fig. 3. Altered fasting and non-fasting lipoprotein composition in *PNPLA3* GG carriers. Volcano plot showing (A) fasting and (B) non-fasting lipoproteins of *PNPLA3* GG (n = 32 (A)/17 (B)) compared with CC carriers (n = 83 (A)/87 (B)). (C) Comparison of absolute concentrations of lipoprotein subgroups and their lipid composition between groups (*PNPLA3* GG *vs*. *PNPLA3* CC). Color coding represents the fold change in mean concentrations between groups. The brighter the red or blue color, the greater the increase or decrease in the absolute concentration of a lipoprotein subfraction in *PNPLA3* GG compared with *PNPLA3* CC, respectively, in both the fasting and non-fasting states. Significant differences in (D) fasting lipoproteins and (E) specific non-fasting lipoproteins between groups (bars represent SD, and bold lines within the box plots represent medians). Levels of significance: *q <0.05; **q <0.01; ***q <0.001; ***q <0.001 (Mann–Whitney *U* test; FDR = 5%). FDR, false discovery rate; PNPLA3, patatin-like phospholipase domain-containing protein.

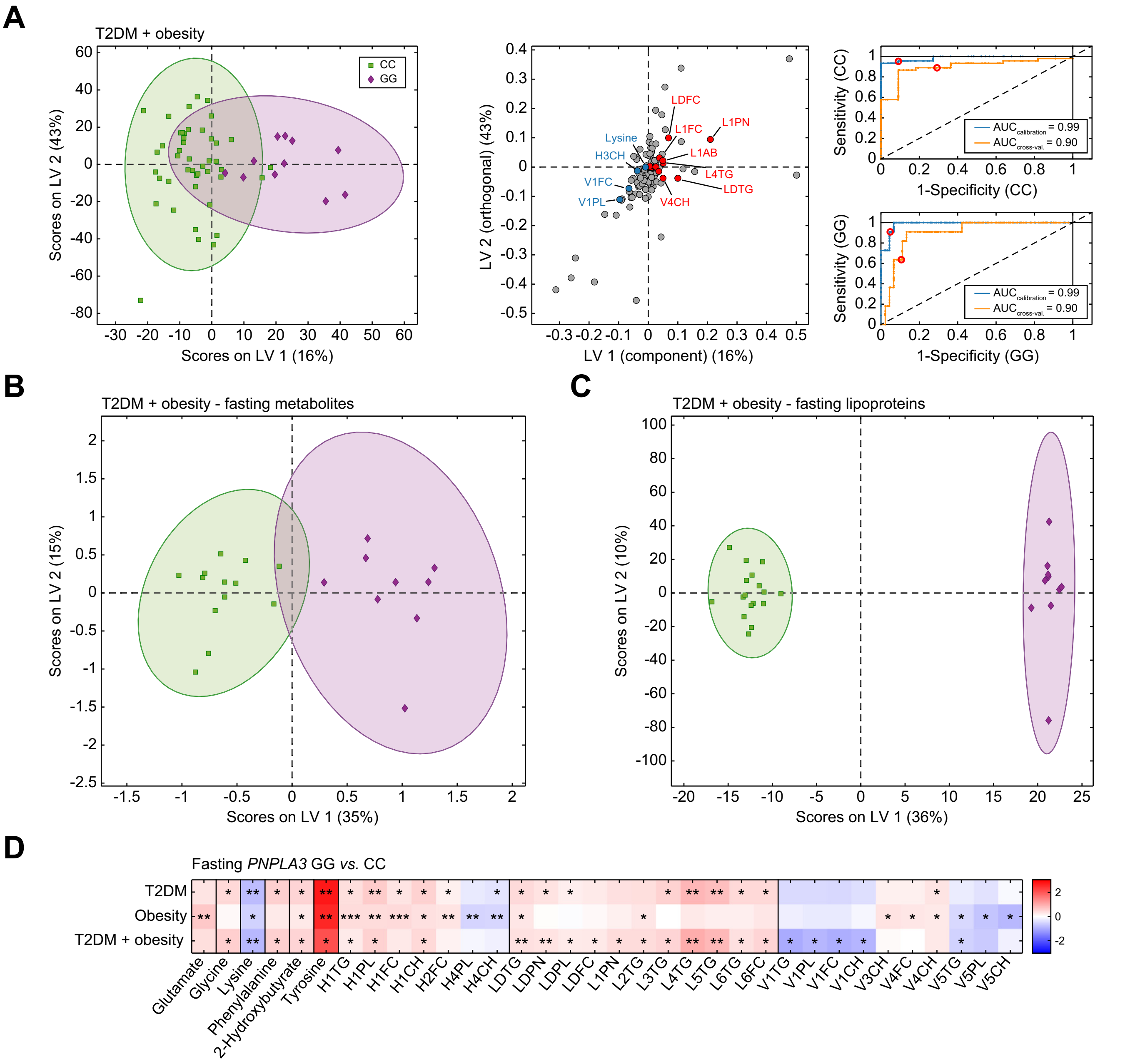

Fig. 4. *PNPLA3* GG carriers with T2DM and obesity show pronounced changes in the metabolome. (A) Distribution of *PNPLA3* GG carriers with T2DM and obesity (n = 11) compared with CC carriers with T2DM and obesity (n = 46) using the O-PLS-DA of metabolites and lipoproteins, with a loading plot showing significant parameters (*q* value <0.05) (red and blue indicate increase and decrease in GG carriers, respectively) and corresponding ROC curves. Separation of *PNPLA3* GG *vs.* CC carriers with T2DM and obesity based on the O-PLS-DA of (B) fasting metabolites and (C) fasting lipoproteins. (D) Comparison of absolute concentrations of fasting metabolites and lipoprotein subgroups and their lipid composition between groups (*PNPLA3* GG *vs. PNPLA3* CC) with T2DM, obesity, or both conditions. Color coding represents the fold change in mean concentrations between groups. The brighter the red or blue color, the greater the increase or decrease in the absolute concentration of parameters in fasting *PNPLA3* GG compared with CC carriers, respectively. Levels of significance: **q* <0.05; ***q* <0.01; ****q* <0.001 (Mann–Whitney *U* test; FDR = 5%). FDR, false discovery rate; O-PLS-DA, orthogonal partial least squares discriminant analysis; PNPLA3, patatin-like phospholipase domain-containing protein; ROC, receiver operating characteristic; T2DM, type 2 diabetes mellitus.

Finally, to determine whether the trend towards higher fibrosis risk observed in the GG carriers of our cohort is attributed to higher proinflammatory markers, we performed an analysis of glycoprotein profiles separated by FIB-4. Consistently, glycoprotein profiles in *PNPLA3* GG and CC carriers showed no discernible differences in the distribution of FIB-4 index values, either above or below 1.3 (Fig. 5B).

Discussion

The *PNPLA3* rs738409 (p.I148M) polymorphism is the most important genetic risk factor associated with MASLD progression to MASH and advanced fibrosis.[26,30] Although underlying mechanisms of the impact of the genotype during this sequence remain incompletely understood, accumulating evidence assigns functional and clinical consequences to GG

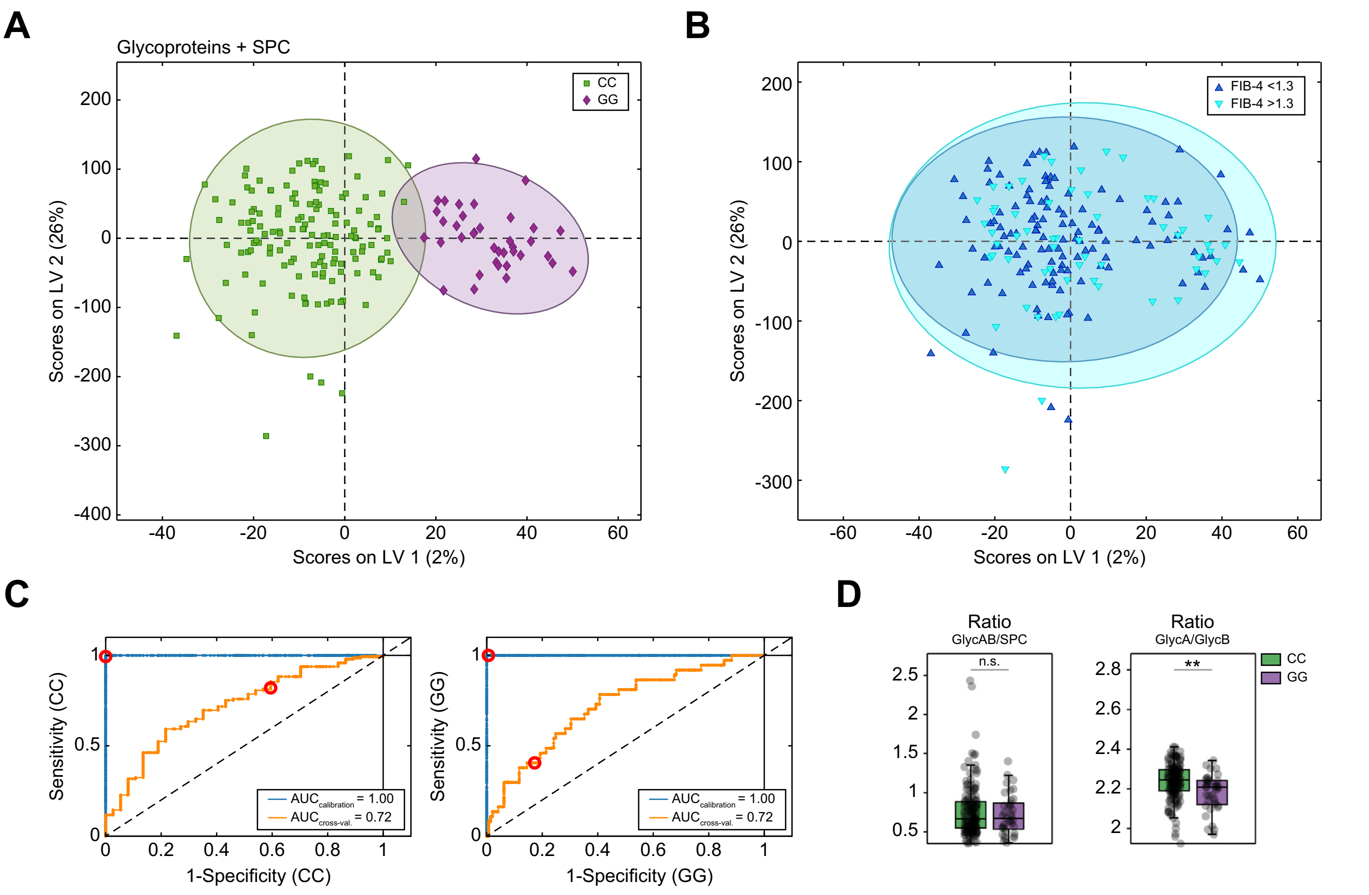

Fig. 5. Variations in glycoprotein and phosphatidylcholine profiles according to *PNPLA3* genotype. (A) Separation of *PNPLA3* GG *vs.* CC carriers based on the O-PLS-DA of glycoproteins and SPC signal. (B) Distribution of patients with FIB-4 <1.3 *vs.* >1.3 within the separation of genotypes based on glycoproteins and SPCs. Levels of significance: $^{**}q$ <0.01 (Mann–Whitney *U* test; FDR = 5%). (C) ROC curves corresponding to O-PLS-DA of glycoproteins and SPCs. (D) Box plots of specific ratios (GlycAB/SPC and GlycA/GlycB) comparing groups (bars represent SD, and bold lines within the box plots represent medians). FDR, false discovery rate; O-PLS-DA, orthogonal partial least squares discriminant analysis; PNPLA3, patatin-like phospholipase domain-containing protein; ROC, receiver operating characteristic; SPC, supramolecular phosphatidylcholine composite signal.

carriers.[11] Findings presented here align with these observations and demonstrate that homozygous *PNPLA3* GG carriers exhibit a distinct metabolic profile compared with CG or CC carriers, as revealed by NMR-proteometabolomics. Our results provide new mechanistic clues and highlight how fasting and non-fasting states exacerbate pathophysiological alterations in GG carriers, emphasizing critical implications for biomarker identification and preventive strategies.

Patients with MASLD carrying at least one 148M allele are predisposed to more severe liver disease, with the risk of being particularly pronounced in homozygous carriers.[26] In the present study, while heterozygous *PNPLA3* CG carriers show elevated ALT levels comparable to GG carriers, their metabolomic profiles are not notably different from non-carriers (CC). In contrast, NMR-proteometabolomics identifies distinct metabolite and lipoprotein signatures in homozygous GG carriers compared with both CC and CG carriers. These findings suggest that a single allelic change in the *PNPLA3* gene is not sufficient to cause systemic changes in the serum metabolome.

Furthermore, *PNPLA3* is known to be a direct target of the insulin-regulated transcription factor sterol regulatory element binding protein-1c (SREBP-1c) and is, therefore, tightly regulated by fasting and feeding. In particular, carbohydrate intake activates SREBP-1c, which transcriptionally induces *PNPLA3*.[15,31] In this case, PNPLA3 148M results in an increase in intrahepatic lipid storage.[11] In turn, low plasma insulin concentrations, such as those observed during overnight fasting, may decrease the expression of *PNPLA3*. Consequently, inhibition of ATGL by PNPLA3 148M upon fasting is expected to cease, reversing the negative impact of the variant on lipolysis of lipid droplet TGs and inducing a pronounced increase in β-oxidation. The latter results in the accumulation of acetyl-CoA in the liver and induces two different molecular traits: either oxidation via the TCA cycle or condensation in the ketogenic pathway to form ketone bodies.[32] Consistent with this, differences in NMR-proteometabolites between *PNPLA3* GG and CC genotypes become increasingly apparent when fasting status is considered, which is a major finding of the presented study.

In particular, we have shown that fasted *PNPLA3* GG carriers have higher levels of succinate, a key metabolite, and glutamate, a product of the TCA cycle. In line with this, recent data revealed higher TCA aconitinate in fasted *PNPLA3* GG carriers, as determined by non-targeted relative quantitative liquid chromatography-tandem mass spectrometry.[33] Alternatively, glutamate could also be catalyzed by blood ALT,[34]

whereas in our study, we did not observe differences in the serum glutamine-to-glutamate ratio or a correlation between glutamate and ALT levels (Fig. S2D and E), indicating that glutamate production is more likely driven by the TCA cycle originating from α-ketoglutarate. Furthermore, it has been shown that patients with MASLD generally exhibit increased fasting β-oxidation but reduced rates of ketogenesis and increased reliance on the TCA cycle, suggesting that this may be necessary to maintain normal ATP production and energy homeostasis in the liver.[35] Conversely, we observed higher fasting ketone bodies in *PNPLA3* GG compared with CC carriers. Luukkonen *et al.*[14] proposed that these changes are, in turn, associated with alterations in the hepatic mitochondrial redox state, thereby inhibiting hepatic citrate synthase flux and limiting citrate export to the cytosol for *de novo* lipogenesis. This further stimulates carnitine palmitoyltransferase-1-mediated entry of fatty acids into the mitochondria, leading to a vicious cycle of increased β-oxidation and mitochondrial dysfunction and ultimately aggravating liver injury.[14] Our results support these findings and extend the mechanistic implications for patients with genetic susceptibility by demonstrating that lysine, an exclusively ketogenic amino acid and a key component of carnitine, is consequently significantly reduced in homozygous *PNPLA3* GG carriers. Moreover, 2-hydroxybutyrate, a by-product of cystathionine cleavage to cysteine that is used for glutathione synthesis to mitigate redox stress, is significantly elevated in *PNPLA3* GG carriers, further indicating an elevated hepatic redox state in these patients.[36]

In the fasting state, increased lipolysis of lipid droplet TGs is also evidenced by a high TG load across several LDL subtypes, with additional elevations of TGs within larger HDL particles (HDL-1 and HDL-2). Previous studies also reported smaller VLDLs and larger LDLs in fasted *PNPLA3* GG carriers, suggesting a potential antihyperlipidemic effect of the variant.[37–39] However, our data demonstrate that these effects are highly dependent on fasting and non-fasting states, which should raise caution against generalization. In addition, fasted *PNPLA3* GG carriers exhibit higher levels of VLDL-3 and VLDL-4 CH, which also causes higher levels of IDL-CH and even IDL particle number. Notably, the latter may serve as a novel and distinctive biomarker identifying fasted homozygous *PNPLA3* GG carriers, offering a practical application for clinical use.

Importantly, in the non-fasting state, PNPLA3 148M indirectly inhibits ATGL, resulting in a tendency toward decreased transport of TG, PL, and CH via VLDL-1 particles from the liver to the periphery, along with a significant increase in VLDL-5 PL and CH. Consistent with this, we observed significantly higher levels of small LDL-4 particles in the non-fasting state, probably resulting from the overall shift in lipoprotein composition. Moreover, a recent study indicates that the effects of the PNPLA3 I148M variant on hepatic steatosis may arise not only from its gain of function as an ATGL inhibitor but also from its loss of function in facilitating TG secretion under lipogenic conditions, suggesting a potentially broader interplay.[40]

Low-grade systemic inflammation is a key component of metabolic diseases, including MASLD.[41] Recent studies have highlighted the role of glycoprotein NMR markers in inflammatory and autoimmune diseases, as GlycA and GlycB have been linked to systemic inflammation, cardiovascular disease, and T2DM.[28,42,43] In our cohort, we do not detect signs of increased inflammation based on glycoprotein SPC profiles, as the GlycAB to SPC ratio remains unchanged.[25,29,44] Although *PNPLA3* GG carriers have higher FIB-4 levels, the glycoprotein profiles show no significant differences in the distribution of FIB-4 values, whether above or below 1.3. This observation may be attributed to the fact that the mean FIB-4 scores across all groups remain closely clustered and within the intermediate FIB-4 risk category. Moreover, a recent study assessed glycoproteins in patients with MASLD and found associations with the fatty liver index but weaker correlations with FIB-4, which might be explained by the observation that FIB-4 is more indicative of fibrosis than inflammation.[45] In addition, *PNPLA3* homozygosity also reduces platelet counts in general, potentially affecting the diagnostic accuracy of FIB-4.[46] Likewise, in our cohort, the platelet count in GG carriers is not only different from that in CC carriers but also significantly lower than that in heterozygous CG carriers, which subsequently influences the FIB-4 index (Table S1). Finally, it is important to emphasize that although NMR is a reliable quantitative method for assessing global glycosylation states, it is limited in its ability to identify the specific proteins involved. Therefore, although the glycoprotein differences observed between *PNPLA3* genotypes were compelling, their exact sources remain undefined.

Furthermore, tyrosine levels are elevated in *PNPLA3* GG carriers, where tyrosine primarily serves as a precursor for catecholamines and thyroxine. Recently, Huang *et al.*[47] demonstrated that higher fasting tyrosine levels correlate with more severe disease, as indicated by the new MASH prediction score. In our study, *PNPLA3* GG carriers exhibit higher MASH scores in the fasting state compared with CC carriers, showing a linear correlation between tyrosine levels and FIB-4 (Fig. S5A and B). Notably, there is no correlation between lysine levels or IDL particle number and FIB-4, confirming that these findings are attributable to the *PNPLA3* GG genotype and not to a higher fibrosis degree (Fig. 5C and D). In addition, ANCOVA adjusted for ALT, AST, platelet count, and *PNPLA3* genotype (GG *vs*. CC) in fasting patients demonstrated a persistent trend towards lower lysine and significantly higher IDL levels in *PNPLA3* GG carriers, although the latter could be additionally influenced by ALT levels, but with a smaller effect size than genotype (Table S2).

Moreover, obesity was reported to be an amplifier of the genetic risk, and this effect may be mediated by insulin resistance in *PNPLA3* GG carriers.[48,49] Recently, patients with MASLD carrying the *PNPLA3* rs738409 polymorphism have been shown to be as insulin resistant as patients with MASLD without the polymorphism at the level of liver, muscle, and adipose tissue.[50] Consistent with this, we demonstrated a better separation of the metabolome between *PNPLA3* GG and CC carriers in patients with T2DM and obesity compared with the total cohort. In particular, the effects on impaired lipid transport from the liver, specifically reduced VLDL-1 levels, are already apparent in the fasting state, suggesting that pronounced insulin resistance may consistently enhance the effect of PNPLA3 148M.

In conclusion, our study emphasizes that the *PNPLA3* rs738409 (p.I148M) polymorphism most likely exerts its deleterious effects via the metabolic switch between fasting and feeding, as our data demonstrate that fasting is an important determinant to unmask the GG impact. The variant not only accelerates intrahepatic TG accumulation in the non-fasted state and disrupts serum lipoprotein composition but also

triggers excessive lipid oxidation during overnight fasting, affecting various metabolic pathways such as the TCA cycle, ketogenesis, and subsequent mitochondrial function, thus most likely causing cellular toxicity in *PNPLA3* GG carriers. Key differences associated with these alterations include reduced lysine levels and a marked increase in fasting IDL particle number. Our findings reveal a dichotomous pattern in *PNPLA3* GG carriers that fuels a vicious cycle of fasting and non-fasting and underscore the need for preventive strategies at an early disease stage.

Affiliations

[1]*Department of Medicine I, University Hospital Schleswig-Holstein, Lübeck, Germany;* [2]*Institute of Chemistry and Metabolomics, University of Lübeck, Lübeck, Germany;* [3]*Institute of Clinical Chemistry and Laboratory Medicine, Carl von Ossietzky University, Oldenburg, Germany;* [4]*German Center for Diabetes Research (DZD), Neuherberg, Munich, Germany;* [5]*Department of Surgery, University Hospital Schleswig-Holstein, Lübeck, Germany;* [6]*Department of Medicine II, University Hospital Saarland, Homburg, Germany;* [7]*Department of Medicine II, University Hospital Würzburg, Bayern, Würzburg, Germany;* [8]*Novo Nordisk Pharma GmbH, Mainz, Germany;* [9]*Department of Gastroenterology, Hepatology and Transplant Medicine, Medical Faculty, University of Duisburg-Essen, Essen, Germany*

Abbreviations

ALT, alanine aminotransferase; Apo, apolipoprotein; AST, aspartate aminotransferase; ATGL, adipose triglyceride lipase; ATP, adenosine triphosphate, CAP, controlled attenuation parameter; CH, cholesterol; FC, free cholesterol; FDR, false discovery rate; GlycA, glycoprotein A signal originating from sialic acid on N-glycans bound to acute-phase inflammation proteins; GlycB, glycoprotein B signal originating from N-acetylglucosamine on N-glycans bound to acute-phase inflammation proteins; IDL, intermediate-density lipoprotein; MASH, metabolic dysfunction-associated steatohepatitis; MASLD, metabolic dysfunction-associated steatotic liver disease; NMR, nuclear magnetic resonance; O-PLS-DA, orthogonal partial least squares discriminant analysis; PL, phospholipid; PNPLA3, patatin-like phospholipase domain-containing protein; SGLT-2, sodium-glucose cotransporter-2; SOP, standard operating procedure; SPC, supramolecular phosphatidylcholine composite signal; SREBP-1c, insulin-regulated transcription factor sterol regulatory element binding protein-1c; T2DM, type 2 diabetes mellitus; TCA, tricarboxylic acid; TG, triglycerides; VCTE, vibration-controlled transient elastography.

Financial support

No funding was received for the current project. LJ received financial support from the German Society of Internal Medicine (DGIM). We also thank the Else Kröner-Fresenius Foundation (EKFS) for financial support. LR, AM, and ULG thank the VDI programme of the BMBF (grant no. 13GW0592E, REMOLCO) and the EC for support under the ONCOSCREEN programme (HORIZON-MISS-2021-CANCER-02, grant no. 101097036) for funding. We also thank the University of Lübeck, Germany, for the provision of facilities and resources that made this research possible.

Conflicts of interest

JUM received funding from NovoNordisk, Abbvie, and Merz.

Please refer to the accompanying ICMJE disclosure forms for further details.

Authors' contributions

Contributed to the planning and design of the study: LJ, DC, MK, AG, JMS, SMM, ULG, JUM. Collected patient data: LJ, BF, LH, FS, SM, AS, MR. Performed *PNPLA3* genotyping: SNW, MK. Conducted NMR-proteometabolomics: LR, AKR, AM. Performed statistical analysis and data interpretation: LJ, LR, DC, AM. Wrote the first draft of the manuscript: LJ, JUM. Approved the final version of the manuscript: all co-authors.

Data availability statement

Data are available on request from the corresponding author. Prior approval from the University of Lübeck may be required.

Declaration of Generative AI and AI-assisted technologies in the writing process

During the preparation of this work the authors used ChatGPT and DeepL to improve readability and language. After using these services, the authors reviewed and edited the content as needed and take full responsibility for the content of the publication.

Acknowledgements

We are grateful for the excellent technical assistance by Monika Herr. The graphical abstract and Fig. 1A were created in BioRender. Jegodzinski, L. (2025) https://BioRender.com/5pnfdjd.

Supplementary data

Supplementary data to this article can be found online at https://doi.org/10.1016/j.jhepr.2025.101450.

References

Author names in bold designate shared co-first authorship

[1] Younossi ZM, Golabi P, de Avila L, et al. The global epidemiology of NAFLD and NASH in patients with type 2 diabetes: a systematic review and meta-analysis. J Hepatol 2019;71:793–801.

[2] Younossi ZM, Golabi P, Paik JM, et al. The global epidemiology of nonalcoholic fatty liver disease (NAFLD) and nonalcoholic steatohepatitis (NASH): a systematic review. Hepatology 2023;77:1335–1347.

[3] Loomba R, Friedman SL, Shulman GI. Mechanisms and disease consequences of nonalcoholic fatty liver disease. Cell 2021;184:2537–2564.

[4] Stefan N, Cusi K. A global view of the interplay between non-alcoholic fatty liver disease and diabetes. Lancet Diabetes Endocrinol 2022;10:284–296.

[5] Sanyal AJ. Past, present and future perspectives in nonalcoholic fatty liver disease. Nat Rev Gastroenterol Hepatol 2019;16:377–386.

[6] Ericson E, Bergenholm L, Andréasson A-C, et al. Hepatic patatin-like phospholipase domain-containing 3 levels are increased in I148M risk allele carriers and correlate with NAFLD in humans. Hepatol Commun 2022;6:2689–2701.

[7] He S, McPhaul C, Li JZ, et al. A sequence variation (I148M) in PNPLA3 associated with nonalcoholic fatty liver disease disrupts triglyceride hydrolysis. J Biol Chem 2010;285:6706–6715.

[8] **Pirazzi C**, **Valenti L**, **Motta BM**, et al. PNPLA3 has retinyl-palmitate lipase activity in human hepatic stellate cells. Hum Mol Genet 2014;23:4077–4085.

[9] Chalasani N, Vilar-Gomez E, Loomba R, et al. PNPLA3 rs738409, age, diabetes, sex, and advanced fibrosis jointly contribute to the risk of major adverse liver outcomes in metabolic dysfunction-associated steatotic liver disease. Hepatology 2024;80:1212–1226.

[10] Wang Y, Kory N, BasuRay S, et al. PNPLA3, CGI-58, and inhibition of hepatic triglyceride hydrolysis in mice. Hepatology 2019;69:2427–2441.

[11] **Wang Y**, **Hong S**, Hudson H, et al. PNPLA3(148M) is a gain-of-function mutation that promotes hepatic steatosis by inhibiting ATGL-mediated triglyceride hydrolysis. J Hepatol 2025;82:871–881.

[12] BasuRay S, Smagris E, Cohen JC. et al. The PNPLA3 variant associated with fatty liver disease (I148M) accumulates on lipid droplets by evading ubiquitylation. Hepatology 2017;66:1111–1124.

[13] **Park J**, **Zhao Y**, **Zhang F**, et al. IL-6/STAT3 axis dictates the PNPLA3-mediated susceptibility to non-alcoholic fatty liver disease. J Hepatol 2023;78:45–56.

[14] Luukkonen PK, Porthan K, Ahlholm N, et al. The PNPLA3 I148M variant increases ketogenesis and decreases hepatic de novo lipogenesis and mitochondrial function in humans. Cell Metab 2023;35:1887–1896.

[15] Huang Y, He S, Li JZ, et al. A feed-forward loop amplifies nutritional regulation of PNPLA3. Proc Natl Acad Sci U S A 2010;107:7892–7897.

[16] Rinella ME, Lazarus JV, Ratziu V, et al. A multisociety Delphi consensus statement on new fatty liver disease nomenclature. J Hepatol 2023;79:1542–1556.

[17] European Association for the Study of the Liver. EASL–EASD–EASO Clinical Practice Guidelines on the management of metabolic dysfunction-associated steatotic liver disease (MASLD). J Hepatol 2024;81:492–542.

[18] Ferrannini E, Baldi S, Frascerra S, et al. Shift to fatty substrate utilization in response to sodium–glucose cotransporter 2 inhibition in subjects without diabetes and patients with type 2 diabetes. Diabetes 2016;65:1190–1195.

[19] Krawczyk M, Rau M, Schattenberg JM, et al. Combined effects of the PNPLA3 rs738409, TM6SF2 rs58542926, and MBOAT7 rs641738 variants on NAFLD severity: a multicenter biopsy-based study1. J Lipid Res 2017;58:247–255.
[20] **Schmelter F**, **Föh B**, Mallagaray A, et al. Metabolic and lipidomic markers differentiate COVID-19 from non-hospitalized and other intensive care patients. Front Mol Biosci 2021;8:737039.
[21] Vehtari A, Mäkinen V-P, Soininen P, et al. A novel Bayesian approach to quantify clinical variables and to determine their spectroscopic counterparts in 1H NMR metabonomic data. BMC Bioinformatics 2007;8:S8.
[22] Wist J, Nitschke P, Conde R, et al. Benchtop proton NMR spectroscopy for high-throughput lipoprotein quantification in human serum and plasma. Anal Chem 2025;97:6399–6409.
[23] Lodge S, Nitschke P, Kimhofer T, et al. Diffusion and relaxation edited proton NMR spectroscopy of plasma reveals a high-fidelity supramolecular biomarker signature of SARS-CoV-2 infection. Anal Chem 2021;93:3976–3986.
[24] Rudolph L, Krellmann R, Castven D, et al. Fast NMR-based assessment of cancer-associated protein glycosylations from serum samples. Anal Chem 2025;97:9367–9377.
[25] Mallagaray A, Rudolph L, Lindloge M, et al. Towards a precise NMR quantification of acute phase inflammation proteins from human serum. Angew Chem Int Ed Engl 2023;62:e202306154.
[26] Carlsson B, Lindén D, Brolén G, et al. Review article: the emerging role of genetics in precision medicine for patients with non-alcoholic steatohepatitis. Aliment Pharmacol Ther 2020;51:1305–1320.
[27] Otvos JD, Shalaurova I, Wolak-Dinsmore J, et al. GlycA: a composite nuclear magnetic resonance biomarker of systemic Inflammation. Clin Chem 2015;61:714–723.
[28] Muhlestein JB, May H, Winegar D, et al. GlycA and GlycB, novel NMR biomarkers of inflammation, strongly predict future cardiovascular events, but not the presence of coronary artery disease (CAD), among patients undergoing, coronary angiography: the Intermountain Heart Collaborative Study. J Am Coll Cardiol 2014;63:A1389.
[29] Masuda R, Lodge S, Whiley L, et al. Exploration of human serum lipoprotein supramolecular phospholipids using statistical heterospectroscopy in n-dimensions (SHY-n): identification of potential cardiovascular risk biomarkers related to SARS-CoV-2 infection. Anal Chem 2022;94:4426–4436.
[30] Unalp-Arida A, Ruhl CE. Patatin-like phospholipase domain-containing protein 3 I148M and liver fat and fibrosis scores predict liver disease mortality in the U.S. population. Hepatology 2020;71:820–834.
[31] Dubuquoy C, Robichon C, Lasnier F, et al. Distinct regulation of adiponutrin/PNPLA3 gene expression by the transcription factors ChREBP and SREBP1c in mouse and human hepatocytes. J Hepatol 2011;55:145–153.
[32] Mooli RGR, Ramakrishnan SK. Emerging role of hepatic ketogenesis in fatty liver disease. Front Physiol 2022;13:946474.
[33] Fernandes Silva L, Vangipurapu J, Oravilahti A. et al. Plasma metabolite signatures in male carriers of genetic variants associated with non-Alcoholic fatty liver disease. Metabolites 2023;13:267.
[34] Kamada Y, Hashimoto R, Yamamori H, et al. Impact of plasma transaminase levels on the peripheral blood glutamate levels and memory functions in healthy subjects. BBA Clin 2016;5:101–107.
[35] Fu X, Fletcher JA, Deja S, et al. Persistent fasting lipogenesis links impaired ketogenesis with citrate synthesis in humans with nonalcoholic fatty liver. J Clin Invest 2023;133:e167442.
[36] **Lord R**, **Bralley J**. Clinical applications of urinary organic acids. Part 1: detoxification markers. Altern Med Rev 2008;13:205–215.
[37] Jiang ZG, Tapper EB, Kim M, et al. Genetic determinants of circulating lipoproteins in nonalcoholic fatty liver disease. J Clin Gastroenterol 2018;52:444–451.
[38] **Luukkonen PK**, **Qadri S**, Lehtimäki TE, et al. The PNPLA3-I148M variant confers an antiatherogenic lipid profile in insulin-resistant patients. J Clin Endocrinol Metab 2021;106:e300–e315.
[39] Ahmed A, Cule M, Bell JD, et al. Differing genetic variants associated with liver fat and their contrasting relationships with cardiovascular diseases and cancer. J Hepatol 2024;81:921–929.
[40] Johnson SM, Bao H, McMahon CE, et al. PNPLA3 is a triglyceride lipase that mobilizes polyunsaturated fatty acids to facilitate hepatic secretion of large-sized very low-density lipoprotein. Nat Commun 2024;15:4847.
[41] Targher G, Byrne CD, Tilg H. MASLD: a systemic metabolic disorder with cardiovascular and malignant complications. Gut 2024;73:691.
[42] Connelly MA, Gruppen EG, Wolak-Dinsmore J, et al. GlycA, a marker of acute phase glycoproteins, and the risk of incident type 2 diabetes mellitus: PREVEND study. Clin Chim Acta 2016;452:10–17.
[43] Connelly MA, Otvos JD, Shalaurova I, et al. GlycA, a novel biomarker of systemic inflammation and cardiovascular disease risk. J Transl Med 2017;15:219.
[44] Fuertes-Martín R, Correig X, Vallvé J-C, et al. Human serum/plasma glycoprotein analysis by 1H-NMR, an emerging method of inflammatory assessment. J Clin Med 2020;9:354.
[45] Moreno-Vedia J, Rosales R, Ozcariz E, et al. Triglyceride-rich lipoproteins and glycoprotein A and B assessed by 1H-NMR in metabolic-associated fatty liver disease. Front Endocrinol (Lausanne) 2022;12:775677.
[46] Meroni M, Dongiovanni P. PNPLA3 rs738409 genetic variant inversely correlates with platelet count, thereby affecting the performance of noninvasive scores of hepatic fibrosis. Int J Mol Sci 2023;24:15046.
[47] **Huang Q**, **Qadri SF**, **Bian H**, et al. A metabolome-derived score predicts metabolic dysfunction-associated steatohepatitis and mortality from liver disease. J Hepatol 2025;82:781–793.
[48] Barata L, Feitosa MF, Bielak LF, et al. Insulin resistance exacerbates genetic predisposition to nonalcoholic fatty liver disease in individuals without diabetes. Hepatol Commun 2019;3:894–907.
[49] Stender S, Kozlitina J, Nordestgaard BG, et al. Adiposity amplifies the genetic risk of fatty liver disease conferred by multiple loci. Nat Genet 2017;49:842–847.
[50] Bril F, Kalavalapalli S, Lomonaco R, et al. Insulin resistance is an integral feature of MASLD even in the presence of PNPLA3 variants. JHEP Rep 2024;6:101092.

Keywords: PNPLA3; Fasting; MASLD; NMR-proteometabolomics; Lipoproteins.
Received 9 March 2025; received in revised form 26 April 2025; accepted 2 May 2025; Available online 10 May 2025

Journal of Hepatology, Volume *7*

Supplemental information

PNPLA3 I148M variant links to adverse metabolic traits in MASLD during fasting and feeding

Lina Jegodzinski, Lorena Rudolph, Darko Castven, Friedhelm Sayk, Ashok Kumar Rout, Bandik Föh, Laura Hölzen, Svenja Meyhöfer, Andrea Schenk, Susanne N. Weber, Monika Rau, Sebastian M. Meyhöfer, Jörn M. Schattenberg, Marcin Krawczyk, Andreas Geier, Alvaro Mallagaray, Ulrich L. Günther, and Jens U. Marquardt

PNPLA3 I148M variant links to adverse metabolic traits in MASLD during fasting and feeding

Lina Jegodzinski, Lorena Rudolph, Darko Castven, Friedhelm Sayk, Ashok Kumar Rout, Bandik Föh, Laura Hölzen, Svenja Meyhöfer, Andrea Schenk, Susanne N. Weber, Monika Rau, Sebastian M. Meyhöfer, Jörn M. Schattenberg, Marcin Krawczyk, Andreas Geier, Alvaro Mallagaray, Ulrich L. Günther, Jens U. Marquardt

Table of contents

Supplementary methods……………………………………………………………………2
Fig. S1………………………………………………………………………………………5
Fig. S2………………………………………………………………………………………7
Fig. S3………………………………………………………………………………………8
Fig. S4………………………………………………………………………………………10
Fig. S5………………………………………………………………………………………11
Fig. S6………………………………………………………………………………………12
Table S1……………………………………………………………………………………14
Table S2……………………………………………………………………………………15
Supplementary references……………………………………………………………16

Supplementary methods

NMR-Proteometabolomics

Sample preparation

Samples were prepared following a previously described standard operating procedure (SOP)[1]. Briefly, frozen serum aliquots were thawed at room temperature for a maximum of 30 minutes. Serum and NMR buffer (75 mM sodium phosphate buffer pH 7.40, 20% deuterium oxide, 4.8 mM 3-trimethyl-silyl-[2,2,3,3-$^{2}H_4$]propionic acid or TMSP-d4 and 0.04% NaN_3) were 1:1 homogenized, and 600 µL of the well-mixed sample were transferred to a 5 mm NMR tube. The tubes were cooled at 279 K in an automated SampleJet™ until measurement. Spectra were acquired within a maximum of 24 h from sample preparation.

NMR measurements

All NMR experiments were performed on a Bruker Avance III HD 600 MHz NMR spectrometer equipped with a TXI room temperature probe and a Bruker SampleJet™ automatic sample exchanger with sample storage set at 6 °C. All experiments were acquired at 310 K. Temperature precision, quantification accuracy, water suppression performance and gradient profiles were tested and calibrated when required on a daily basis following Bruker´s commercial SOPs. All NMR experiments were acquired in full automation. In all the cases the carrier was placed at the center of the water signal and the receiving gain was set to 90.5. **^{1}H-NOESY** experiments were acquired using the standard pulse sequence *noesygppr1d* from Bruker library with 32 scans (4m 3s), 96k points, 30 ppm spectral width and 4 s inter-scan delay. **^{1}H-CPMG** experiments were acquired using the standard pulse sequence *cpmgpr1d* from Bruker library with 32 scans

(4m 18s), 96k points, 30 ppm spectral width, 4 s inter-scan relaxation delay, 0.3 ms echo time for a total of 78 ms T_2 relaxation time.

For the processing of these two experiments, FIDs were zero filled to 128k points and multiplied by an exponential decay window function to obtain a line broadening of 0.3 Hz. After Fourier transform, spectra were calibrated to the TMSP-d4 signal at 0 ppm and zero order phase corrected. Bruker Quantification in Plasma/Serum (B.I.Quant-PS 2.0.0) and Bruker IVDr Lipoprotein Subclass Analysis (B.I.-LISA) were used to automatically quantify 39 metabolites (+2 technical additives) and 112 lipoprotein parameters, including very-low-density lipoprotein (VLDL), intermediate-density lipoprotein (IDL), low-density lipoprotein (LDL), and high-density lipoprotein (HDL). In addition, several subfractions of triglycerides (TG), cholesterol (CH), free cholesterol (FC), phospholipids (PL), and apolipoproteins (Apo) were calculated. The LDL was categorized into six subclasses based on specific density ranges: LDL-1 (1.019–1.031), LDL-2 (1.031–1.034), LDL-3 (1.034–1.037), LDL-4 (1.037–1.040), LDL-5 (1.040–1.044), and LDL-6 (1.044–1.063).

NMR glycoslation profiles from circulating acute-phase proteins were derived from **selTOCSY** and **JEDI-PGPE** experiments[2,3]. **SelTOCSY** spectra were acquired using the standard pulse sequence *selmlgp.2* from Bruker library with 32k points and 30 ppm spectral width, 0.3 ms inter-scan relaxation delay and 50 ms TOCSY mixing time. Selective irradiation was achieved using a Gaussian-shaped Gaus1.1000 pulse. Frequency for selective pulse, pulse length and number of scans were as follows: When centered at 3.7 ppm (selTOCSY-3.7), the pulse had a length of 8 ms and 600 scans (13m 8s) were acquired. When centered at 4.3 ppm (selTOCSY-4.3), the pulse had a length of 9 ms and 312 scans (6m 52s) were acquired. When centered at 4.85 ppm (selTOCSY-4.85), the pulse length was set to 25 ms and 600 scans (13m 18s) were acquired. FIDs

were zero-filled to 128k points and multiplied by an exponential decay window function to obtain a line broadening of 0.3 Hz. **JEDI-PGPE** experiments were acquired using the standard pulse sequence *zgpezf.ivdr* from Bruker library with 32 scans (2m 19s), 96k points, 30 ppm spectral width, 1 s inter-scan relaxation delay, the gradient shaped pulses were set to 2.5 ms and 80% gradient strength (from a maximum nominal gradient strength of 48.15 G/cm), the total T_2 relaxation time was 110 ms and the Eddy current time delay was set to 5 ms. In both cases spectra were Fourier transformed, calibrated, zero-order phase corrected, baseline corrected, and lineshape fitted to quantify the phosphatidylcholine concentration from the SPC signal (extracted from PGPE-JEDI experiment) and from SPE-1 and SPE-2 signals (extracted from selTOCSY-3.7 and selTOCSY-4.3, respectively), and to obtain glycosylation profiles[4].

Additionally, **Fig. S6** shows examples of lineshape fitting of representative NMR spectra.

Supplementary figures

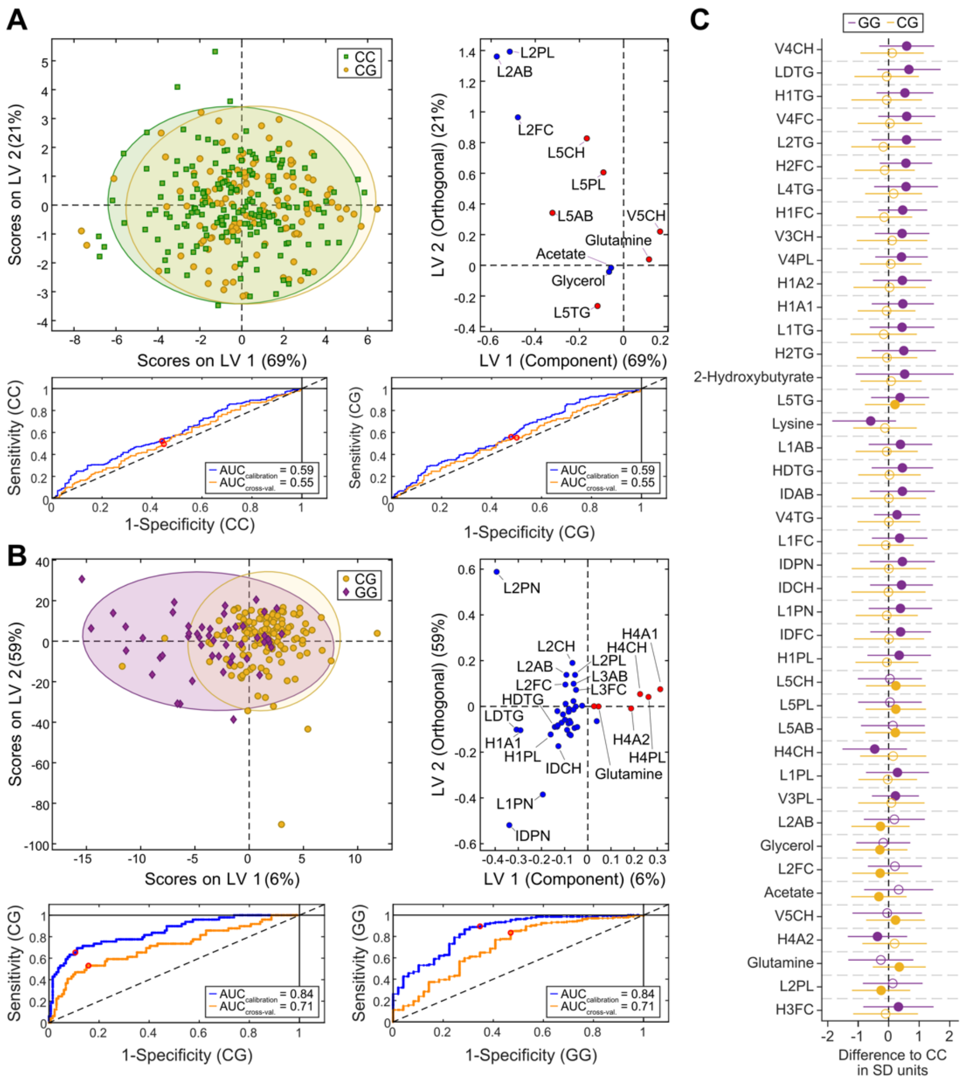

Fig. S1: NMR metabolite and lipoprotein analysis based on *PNPLA3* genotype. (A) Distribution of *PNPLA3* CG *vs.* CC carriers and (B) *PNPLA3* CG *vs.* GG carriers based on the O-PLS-DA of metabolites and lipoproteins of parameters with significant differences between the groups (q-value <0.05), loading plot of significant parameters (red and blue indicate increase and decrease in CG or GG carriers, respectively) and corresponding ROC curves**.** (C) Forest plot showing significant changes in metabolites

and lipoproteins of either CG or GG carriers, both with CC carriers as reference (middle line shows reference mean, while circles on horizontal axes show changes scaled by standard deviations (SD)). Statistically significant differences are indicated by filled circles (determined using a false discovery rate (FDR) of 5%).

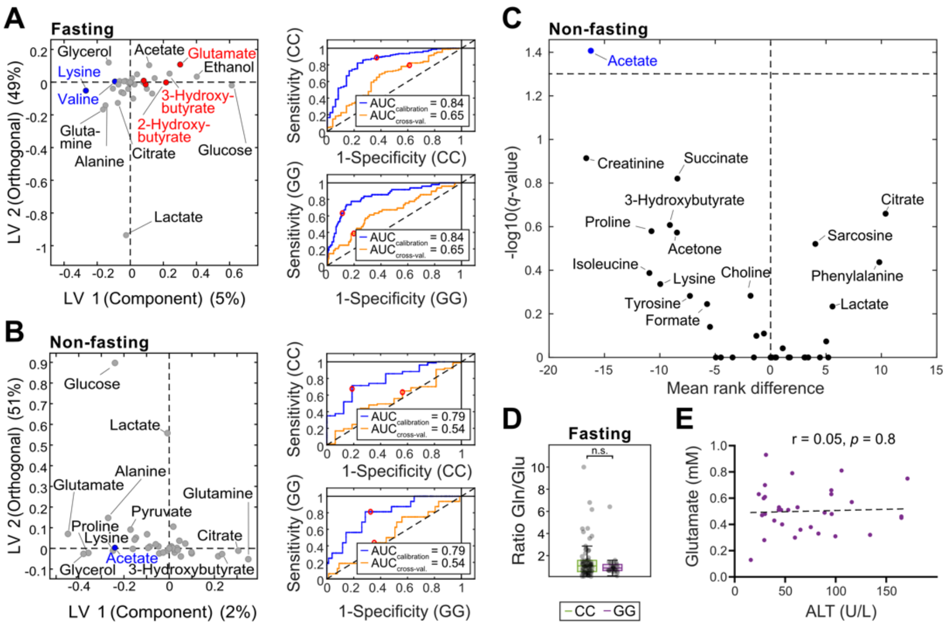

Fig. S2: Fasting and non-fasting metabolite differences between *PNPLA3* GG and CC genotypes. (A) Loading plots and corresponding ROC curves of O-PLS-DAs of fasting and (B) non-fasting metabolites between *PNPLA3* GG and CC carriers. (C) Vulcano plot illustrating non-fasting metabolites of *PNPLA3* GG carriers compared to CC carriers. (D) Box plot of fasting glutamine (Gln)-to-glutamate (Glu) ratio between groups (bars represent SD and bold lines within box plots represent medians; Mann-Whitney U test; false discovery rate (FDR): 5%). (E) Correlation between glutamate and ALT levels in fasting PNPLA3 GG carriers (two-tailed Pearson correlation test).

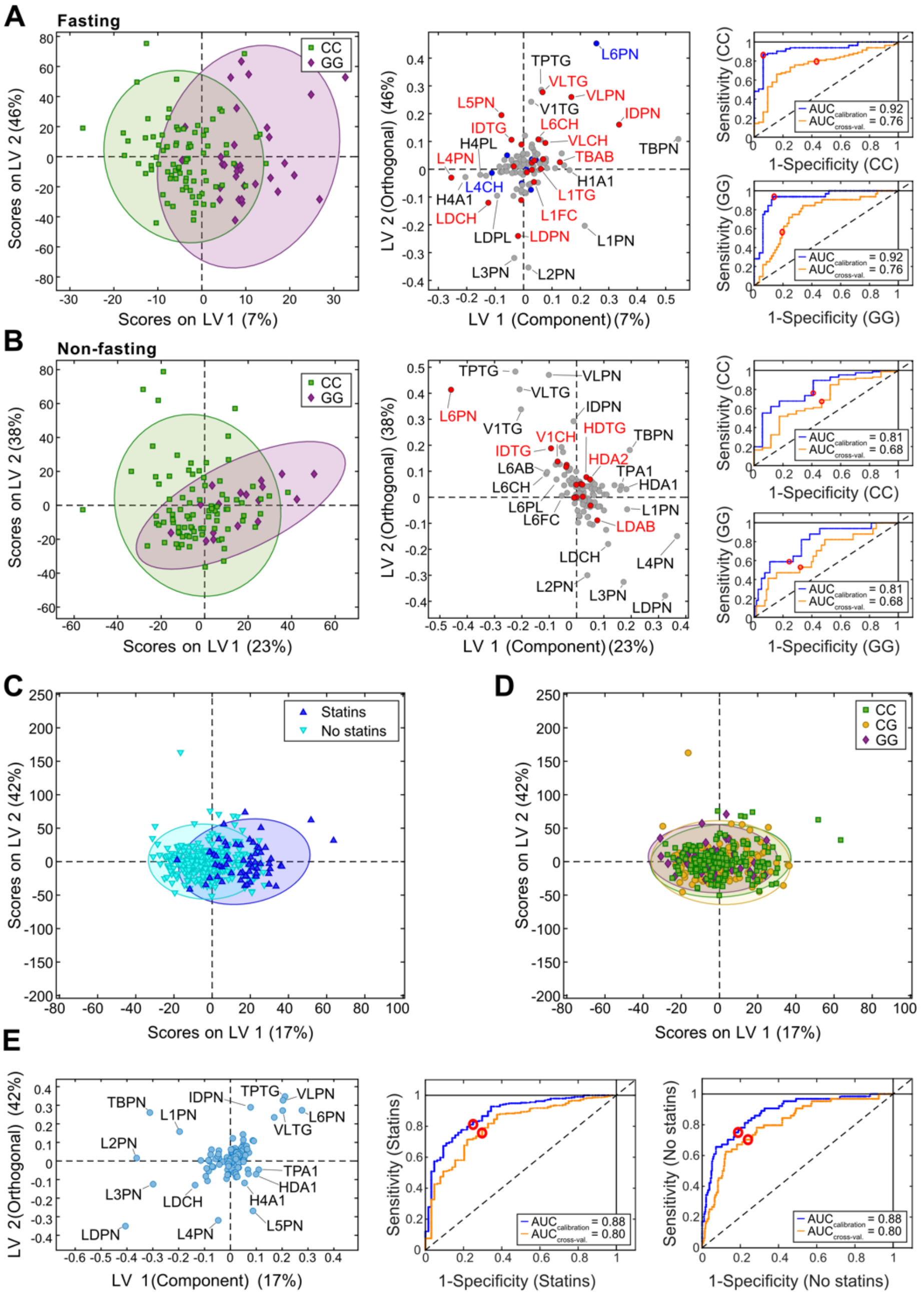

Fig. S3: Remarkable variation in fasting and non-fasting lipoproteins in homozygous *PNPLA3* GG carriers. (A) Separation of *PNPLA3* GG *vs.* CC carriers using O-PLS-DA of fasting and (B) non-fasting lipoproteins, loading plots and corresponding ROC curves**.** (C) Separation of patients with and without statin use based on O-PLS-DA

of lipoproteins and (D) distribution of genotypes (CC, CG and GG) within this separation.

(E) Loading plot and corresponding ROC curves of O-PLS-DA.

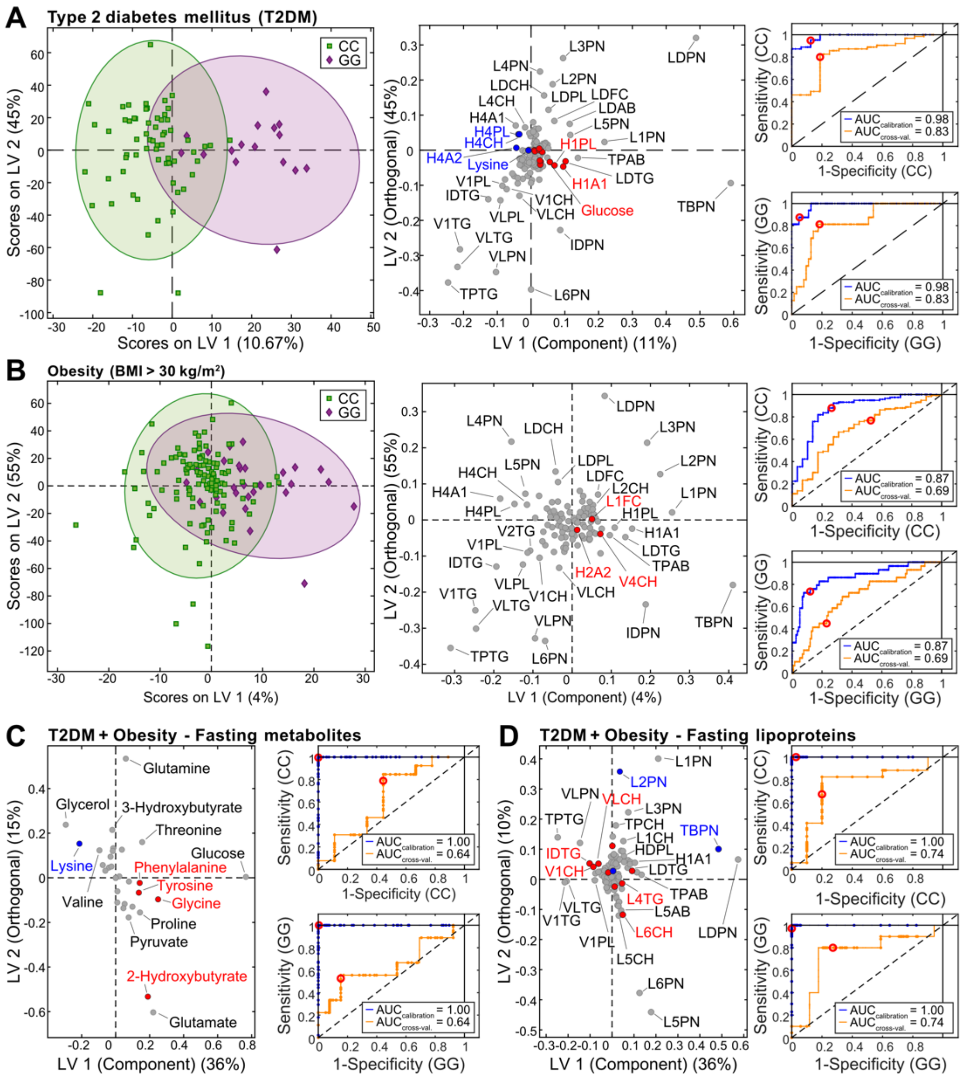

Fig. S4: Type 2 diabetes mellitus (T2DM) and obesity amplify *PNPLA3* GG and CC differentiation. (A) Distribution of *PNPLA3* GG *vs.* CC carriers with T2DM and in (B) of *PNPLA3* GG *vs.* CC carriers with obesity using O-PLS-DA of metabolites and lipoproteins, loading plots highlighting significant parameters (*q*-value <0.05) and corresponding ROC curves. (C) Loading plots and corresponding ROC curves of fasting metabolites and (D) fasting lipoproteins between *PNPLA3* GG and CC carriers with T2DM and obesity.

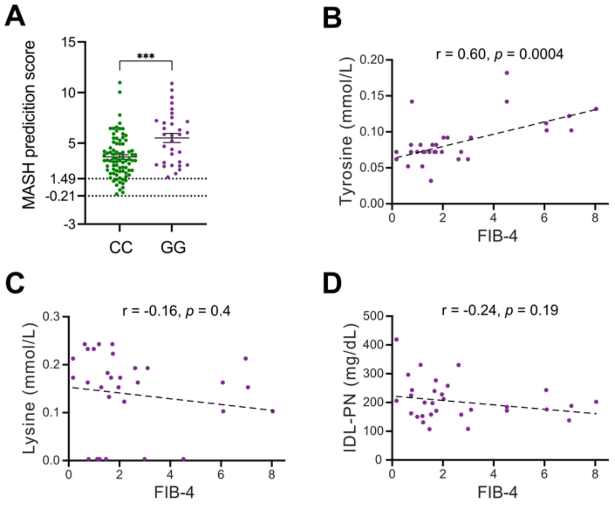

Fig. S5: Correlation between individual parameters and FIB-4. (A) Differences in MASH prediction score between fasting *PNPLA3* GG and CC carriers (mean ± standard error of the mean (SEM)). (B) Correlations between tyrosine, (C) lysine and (D) Intermediate-density lipoprotein particle number (IDL-PN) and FIB-4 values in fasting *PNPLA3* GG carriers (two-tailed Pearson correlation test). ***p <0.001 (Mann-Whitney U test).

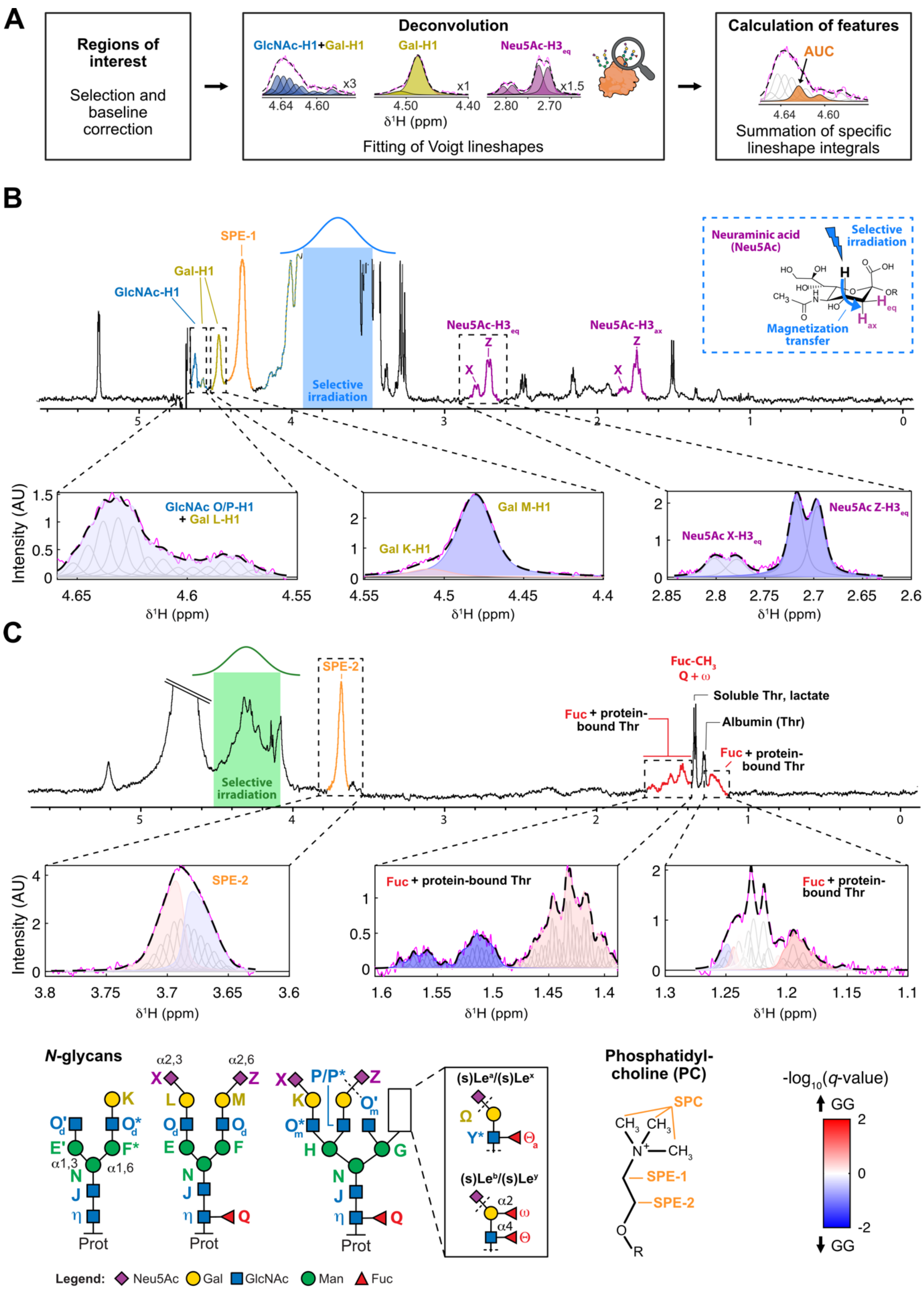
A
Regions of interest
Selection and baseline correction
Deconvolution
GlcNAc-H1+Gal-H1
Gal-H1
Neu5Ac-H3eq
Fitting of Voigt lineshapes
Calculation of features
AUC
Summation of specific lineshape integrals
B
SPE-1
Gal-H1
GlcNAc-H1
Neu5Ac-H3eq
Neu5Ac-H3ax
Selective irradiation
Neuraminic acid (Neu5Ac)
Magnetization transfer
GlcNAc O/P-H1 + Gal L-H1
Gal M-H1
Gal K-H1
Neu5Ac X-H3eq
Neu5Ac Z-H3eq
Intensity (AU)
δ¹H (ppm)
C
SPE-2
Fuc-CH3 Q + ω
Soluble Thr, lactate
Albumin (Thr)
Fuc + protein-bound Thr
N-glycans
Prot
(s)Lea/(s)Lex
(s)Leb/(s)Ley
Phosphatidyl-choline (PC)
SPC
-log10(q-value)
GG
Legend: Neu5Ac Gal GlcNAc Man Fuc

Fig. S6: Examples of lineshape fitting of representative NMR spectra. (A) Workflow for serum glycoprotein profiling using lineshape fitting. (B) Lineshape fitting of selTOCSY-3.7 experiment and (C) selTOCSY-4.3 experiment (cite our Anal. Chem. here). Differences in the area under the fitted curves (AUCs) between *PNPLA3* CC and GG carriers are shown as -$\log_{10}$(*q*-values) in red and blue, whereby red indicates an increase in GG and blue indicates a decrease. The inlet in B demonstrates the principle of selTOCSY, where selected glycan and phosphatidylcholine protons are irradiated to transfer magnetization over three bonds to the target protons, exemplified by selective irradiation of Neu5Ac-H4 and transfer to Neu5Ac-H3.

Supplementary Table

Table S1. Post-hoc analysis of significant patient characteristics based on *PNPLA3* genotype.

	PNPLA3 rs738409		
***p* value[1]**	**CC *vs.* CG**	**CC *vs.* GG**	**CG *vs.* GG**
AST, U/L	0.137	**0.002**	0.142
ALT, U/L	**0.002**	**0.001**	0.790
Platelet count, x10^9/L	0.762	**0.001**	**0.020**
FIB-4	1.000	**0.001**	**<0.001**

ALT, alanine aminotransferase; AST, aspartate aminotransferase.
[1]ANOVA with Bonferroni's adjustment for pairwise comparisons was used. Bold *p* values denote statistical significance at the p <0.05 level.

Table S2. Comparison of Lysine and IDL-particle number (PN) concentrations between fasted *PNPLA3* GG and CC carriers.

Variable	Source	df	Mean square	*F* value	*p* value[1]	partial η²	*r* value
**Lysine** (mmol/L)	AST	1	0.006	0.775	0.380	0.007	0.08
	ALT	1	0.009	1.217	0.272	0.011	0.11
	Platelet count	1	0.014	1.919	0.169	0.017	0.13
	**PNPLA3 genotype**	1	0.023	3.187	0.077	0.028	0.17
**IDL-PN** (mg/dL)	AST	1	1393	0.323	0.571	0.003	0.05
	**ALT**	1	17722	4.112	**0.045**	**0.036**	**0.19**
	Platelet count	1	1117	0.259	0.612	0.002	0.05
	**PNPLA3 genotype**	1	24962	5.792	**0.018**	**0.050**	**0.22**

IDL, intermediate-density lipoprotein; ALT, alanine aminotransferase; AST, aspartate aminotransferase; df, degrees of freedom.

[1]ANCOVA (adjusted for ALT, AST, platelet count and *PNPLA3* genotype (GG *vs.* CC)) was used. Bold *p* values denote statistical significance at the p <0.05 level.

Supplementary references

1. **Schmelter, F, Föh, B**, Mallagaray, A, *et al.* Metabolic and Lipidomic Markers Differentiate COVID-19 From Non-Hospitalized and Other Intensive Care Patients. *Frontiers in Molecular Biosciences* **8,** (2021).

2. Lodge, S, Nitschke, P, Kimhofer, T, *et al.* Diffusion and Relaxation Edited Proton NMR Spectroscopy of Plasma Reveals a High-Fidelity Supramolecular Biomarker Signature of SARS-CoV-2 Infection. *Anal. Chem.* **93,** 3976–3986 (2021).

3. Rudolph, L, Krellmann, R, Castven, D, *et al.* Fast NMR-Based Assessment of Cancer-Associated Protein Glycosylations from Serum Samples. *Anal. Chem.* accepted (2025).

4. Mallagaray, A, Rudolph, L, Lindloge, M, *et al.* Towards a Precise NMR Quantification of Acute Phase Inflammation Proteins from Human Serum. *Angewandte Chemie International Edition* **62,** e202306154 (2023).
